# Comparing diet-related attitudes, perceptions, and behaviors of vegan and omnivorous adults: results from a cross-sectional survey study in Germany

**DOI:** 10.1186/s12889-025-25528-5

**Published:** 2025-12-23

**Authors:** Dan Borzekowski, Emilia Boehm, Natalie Berger, Ann-Kathrin Lindemann, Dino Trescher, Mark Lohmann, Gaby-Fleur Böl

**Affiliations:** Department Risk Communication, German Federal Institute for Risk Assessment (BfR), Berlin, Germany

**Keywords:** Vegan diet, Omnivorous diet, Attitude, Perception, Behavior, Differences

## Abstract

**Background:**

As a well-balanced vegan diet is associated with reduced diet-related health risks, it is in the interest of local authorities to develop appropriate public health interventions to promote such a diet. In order to do so, it is important to understand the psychological characteristics relating to a vegan diet, so that they can be taken into account in the development. This is why the present study explored the diet-related attitudes, perceptions and behaviors of German people who follow a vegan diet and how they differ from those of people who follow an omnivorous diet.

**Methods:**

The data were collected via online questionnaire, using an internet panel. Without being representative of the population, age and gender of the vegan (*n* = 738) and omnivorous (*n* = 824) groups were balanced to ensure comparability in this regard. Statistical tests comprised univariate and multivariate analyses of variance, as well as t-tests and a Mann-Whitney-U-Test.

**Results:**

The main motivations for following a vegan diet were ethical (47%), health (22%) and ecological reasons (14%). The most cited key experience that led to the decision to follow a vegan diet was watching documentaries (73%). 53% of respondents stated that other people had influenced their decision to adopt a vegan diet. Depending on the age of the children, up to 48% of the vegan group and up to 97% of the omnivorous group raise their children on their respective diet. The vegan group perceived significantly lower risks and greater benefits in their own diet (η^2^ = 0.014-0.159). More vegan respondents (86%) reported keeping actively informed on nutrition than did omnivorous respondents (64%), with both groups differing significantly in their perception of the usefulness of several information channels (η^2^ = 0.001-0.075). More vegan respondents (66%) reported taking vitamin B12 supplements than did omnivorous respondents (34%).

**Conclusion:**

The findings are consistent with and build on existing research on cognitive and behavioral patterns related to a vegan diet, while at the same time yielding some additional insights. In particular, the results on significant differences in the risk-benefit perception of a vegan diet, as well as on motivations and influences regarding the decision to follow a vegan diet provide an important basis for the development of public health interventions and a foundation for further studies in this field.

**Supplementary Information:**

The online version contains supplementary material available at 10.1186/s12889-025-25528-5.

## Background

Circulatory diseases were the leading cause of death in Germany in 2021, according to a report by the European Observatory and the OECD [1]. These diseases accounted for 33.3% of all deaths, with ischaemic heart disease being the top cause of mortality at 11.8%, followed by stroke at 5.2%. The report notes that diet-related risks are a major contributor to this mortality, likely through their link to circulatory diseases [1].

A comprehensive meta-analysis from 2018, as well as several more recent studies, concluded that people who follow a vegan diet generally have a lower BMI, smaller waist circumference, lower blood pressure, and better lipid profiles than those on an omnivorous diet [2–7]. These improved metabolic markers may reduce the risk of heart disease, type 2 diabetes, and obesity [4, 8, 9]. Consequently, a vegan diet could potentially lower the occurrence of these major causes of death in Germany. Further benefits associated with a vegan diet include improved kidney function, reduced arthritis symptoms, a lower risk of certain cancers [4, 10, 11], and a distinct gut microbiota profile [12]. Additionally, replacing animal with plant protein has been associated with a lower risk of all-cause mortality [13, 14].

It should be noted here that the advantages of a vegan diet over an omnivorous diet are not exclusive, as the reverse is also true in some respects. The omnivorous diet can, for example, offer advantages in the supply of vitamins B12 and D, as well as essential minerals like iodine [15–17]. However, research indicates that lower dietary intake of vitamin D is a general concern across all dietary groups in children [18]. A study by Turner, Sinclair, and Knez [19], though with a small sample size, found that omnivores consumed insufficient quantities of iron and copper, whereas vegans exhibited significantly lower vitamin B12 levels. Thus, paying attention to sufficient nutrient intake is recommended, irrespective of the dietary pattern followed [20].

This perspective is also reflected by the German Nutrition Society (DGE) in its position paper on vegan diets [21]. While the DGE considers a vegan diet to be healthy, it emphasizes the need for a balanced, well-planned food selection and a vitamin B12 supplement. For vulnerable groups like children, adolescents, pregnant women, nursing mothers, and seniors, the DGE neither recommends nor advises against a vegan diet. In contrast, the Academy of Nutrition and Dietetics considers a vegetarian, including vegan, diet to be appropriate for all life stages, provided it is properly planned [22].

### Research approach

With that in mind, it can be summarized that a well-balanced vegan diet may offer numerous health benefits. It has the potential to significantly reduce diet-related risks and improve public health. Consequently, it could be beneficial for local authorities to convert related scientific evidence into accessible nutrition guidelines and interventions.

For the success of such interventions, understanding the key factors influencing an individual’s dietary choices is essential. Recent qualitative research suggests that an individual’s emotional and value-based relationship with food may substantially influence decision-making, emphasizing factors beyond purely nutritional components [23–25]. This understanding could be effectively guided by a robust theoretical framework, such as the Theory of Planned Behavior (TPB) by Fishbein and Ajzen [26], which is the most widely adopted model for predicting behavior, including dietary habits. The TPB posits that behavior is primarily predicted by intention and perceived behavioral control (PBC). PBC refers to a person’s perception of their ability to perform a behavior. Intention is, in turn, a function of three determinants: attitudes toward the behavior, subjective norms, and PBC. Notably, PBC exerts both a direct effect on the behavior and an indirect effect via intention. Within this framework, the emotional and value-based relationship with food is linked to both attitude and subjective norms, which influence subsequent behavior.

A meta-analysis by McDermott et al. [27] examined the link between TPB variables and dietary patterns. They found that PBC, subjective norms, and attitudes were all significantly associated with the intention to eat healthily. Furthermore, both intention and PBC were significantly linked to actual dietary behavior.

Therefore, when designing an effective public health intervention, it can be valuable to first understand the TPB variables and related psychological constructs in the context of a vegan diet. Specifically, it is pertinent to examine how these variables differ between individuals following vegan and omnivorous diets. This study aims to address this gap by examining the diet-related attitudes, motivations, perceptions, and behaviors of German participants who follow a vegan diet (*n* = 738) and those who follow an omnivorous diet (*n* = 824). The investigation will specifically focus on perceived risks and benefits, nutritional knowledge, information-seeking behavior, health habits, and motivations. The results may provide a resource for the development of public health interventions and inform further research linking TPB and vegan nutrition.

## Materials and methods

### Data collection and participants

Data collection took place in Germany between January 6 and March 1, 2020, as part of a research project at the German Federal Institute for Risk Assessment (BfR) focusing on risk perception towards a vegan diet. A dedicated team of BfR researchers (psychologists and communication scientists) designed the study questionnaire (see Supplementary File 1). Participants were recruited via commercial internet panels (vegan sample: respondi AG, Lightspeed LLC, Dynata; omnivore sample: Dynata). The omnivorous sample was matched to the vegan sample based on age (16–69 years, in categories) and gender (male, female). All participants completed a screening section to confirm their age, gender, and diet before proceeding to the main survey. The initial sample consisted of *n* = 2,059 participants. Data exclusion criteria were applied for incomplete surveys (*n* = 321) and poor-quality responses (*n* = 176). Poor quality was defined by failure to correctly answer validation questions, excessively short completion times (less than 25% of the average), or non-semantic answers to open-ended questions. This process resulted in a final valid sample of 1,562 participants (N_vegan_​=738; N_omnivore​_=824).

### Dietary group categorization

Participants first responded to a screening question by selecting all applicable dietary styles from a provided list (e.g., vegan, vegetarian, omnivore, etc.). Respondents who had selected exactly one of the three main categories (vegan, vegetarian, or omnivore) were categorized immediately. Those selecting multiple or no main categories proceeded to a second screening question, asking them to choose the single style that best described their diet.

- Vegan participants were defined by selecting only “vegan” in the first step or selecting “vegan” in the second step.
- Omnivorous participants were defined by selecting only “omnivore” in the first step or selecting “omnivore” in the second step.

Respondents who ultimately identified as vegetarian were excluded from the main study and directed to a survey exit screen.

### Sociodemographic criteria

The final valid sample consisted of *N* = 1,562 participants with a mean age of 35 years (SD = 12.4), ranging from 16 to 99 years. The sample was composed of 63% women (*n* = 987), 36% men (*n* = 561), and 1% diverse (*n* = 11), defined as belonging neither to the male nor to the female sex. Three participants did not want to answer. The dietary groups were distributed almost equally, with 53% omnivores (*n* = 824) and 47% vegans (*n* = 738).

The omnivorous group (*n* = 824) consisted of 61% women (*n* = 502), 39% men (*n* = 321), and one diverse participant, with a mean age of 35 years (SD = 12.5). The vegan group (*n* = 738) was composed of 66% women (*n* = 485), 33% men (*n* = 240), and 10 diverse participants, with a mean age of 34 years (SD = 12.2). Three vegan participants did not want to answer.

### Survey procedure

The survey was developed and administered online in German and was organized into seven fixed-order sections:

1. Screening (e.g., What is your diet?).
2. Motivations for choosing a vegan diet (e.g., Which of the following was your main motive for deciding to go vegan?).
3. Social environment, pregnancy, and children (e.g., What was your diet during pregnancy?).
4. Nutritional knowledge and information (e.g., Which sources do you use to actively inform yourself about nutrition?).
5. Nutritional habits and consumption behaviors (e.g., How often do you take iron?).
6. Health-related behaviors (e.g., How often do you drink alcohol?).
7. Sociodemographic criteria (e.g., What is your highest level of general education qualification?).

Section 4 on nutritional knowledge and information was presented to all participants using both open- and closed-ended formats. Crucially, the wording of the questions in this section was neutral and did not convey information regarding the nutritional risks or benefits of either dietary pattern.

To maintain respondent engagement and reduce question fatigue, specific filter logic was applied, ensuring that questions relevant only to a subset of participants (e.g., questions on meat consumption levels) were presented exclusively to the respective target groups (e.g., omnivores).

### Data analysis

Descriptive statistics were calculated for all relevant variables. Responses to open-ended questions were coded by the researchers. All analyses were performed using IBM SPSS Statistics 26.

To test for overall group differences between the vegan and omnivorous samples, MANOVAs were conducted. Significant overall differences were further explored using post-hoc tests (univariate ANOVAs or t-tests, unless otherwise stated). A Mann-Whitney U test was performed specifically for differences in physical activity level.

Effect sizes were calculated using Cohen’s d and partial η^2^. Interpretation of these effect sizes followed common thresholds (d=∣0.20∣ small, d=∣0.50∣ medium, d=∣0.80∣ large; η^2^ = 0.01 small, η^2^ = 0.21 medium, η^2^ = 0.35 large [28]). To control the overall probability of a Type 1 error, all p-values were adjusted via Bonferroni correction.

## Results

### Motivations for choosing a vegan diet

Vegan participants (*n* = 738) were asked to indicate their main motivation for adopting a vegan diet. The most frequently indicated motivation was ethical reasons (47%), followed by health reasons (22%), ecological reasons (14%), and social/political reasons (8%), with religious/spiritual reasons (6%) chosen least frequently. Some respondents listed other motivations (2%), while others did not know/did not want to answer (2%).

Vegan respondents who indicated health reasons had been their main motivation for adopting a vegan diet (*n* = 159) indicated food intolerances (28%), obesity (28%), chronic inflammatory diseases (e.g. rheumatoid arthritis, inflammatory bowel disease or chronic skin conditions; 26%), allergies (21%), high blood pressure (18%), diabetes (10%) and cancer (8%) as the main reasons. Some respondents indicated other reasons (11%), while others did not know/did not want to answer (6%).

Some vegan respondents who indicated having been motivated to adopt a vegan diet by a key experience (59% of the vegan sample, *n* = 435) reported this experience to have been related to health reasons (13%) or pregnancy/breastfeeding (3%). However, many indicated an influential role of documentaries (73%), with documentaries on animal rights/animal husbandry in video (25%), radio (4%) or text format (14%) playing a role for some, while others were influenced by documentaries on vegan diets in video (16%), radio (5%) or text format (9%). Some listed other reasons (9%), while others did not know/did not want to answer (2%).

Vegan respondents reporting their decision to adopt a vegan diet was influenced by one or more person(s) (53% of the vegan sample, *n* = 384) rated these persons’ influence(s) on a scale from 1 (no influence at all) to 3 (decided influence). The highest influence reported was from friends or acquaintances (M = 1.98, SD = 0.81), partners (M = 1.86, SD = 0.84), famous or well-known persons (M = 1.83, SD = 0.82), followed by other relatives or children (M = 1.67, SD = 0.83), colleagues (M = 1.59, SD = 0.75), parents or siblings (M = 1.56, SD = 0.76), with persons living in the same shared housing (M = 1.53, SD = 0.77) having the least influence.

Vegan respondents who reported that famous or well-known persons impacted their decision to adopt a vegan diet (*n* = 211) were asked to elaborate on who they were influenced by. Multiple answers were allowed. 26% (*n* = 55) mentioned personalities from the entertainment field, 22% (*n* = 47) mentioned influencers/YouTubers and 6% (*n* = 12) named personalities/experts from the food sector. The remaining respondents mentioned animal rights activists (3%, *n* = 6), persons from the political sector (3%), athletes (3%), health professionals (2%), and others (3%). 44% (*n* = 92) did not know/did not want to answer.

### Respondents’ social environment, pregnancy and children

When prompted regarding the dietary choices of their social environment, some vegan respondents indicated that people who follow a vegan diet were among those living in their household (44%), family members (24%) or friends or acquaintances not living in their household (38%), or within their social environment in a different role (other: 1%), while 19% indicated there were no people who follow a vegan diet in their social environment and 1% did not know/did not want to answer.

Thirty-four percent (*n* = 531) of the total sample reported to have children under 18 years living in their household. These participants were asked about their children’s diets to further examine how their own nutritional styles translate to their children’s. Table 1 gives an overview regarding the diets of the respondents’ children in different age categories, each presented for the vegan and omnivorous group.

**Table 1:** This table presents the number (*n*) of vegan and omnivorous participants who reported to have children aged < 1 year, 1–2 years, 3–6 years, 7–10 years, 11–13 years, and 14–17 years and their respective proportions relative to the total of vegan/omnivorous participants who reported to have children

	Vegan Participants (*n* = 738)		Omnivorous Participants (*n* = 824)	
	n	%	n	%
*Children*	251	34	280	34
*Children aged < 1 year*				
Number of Children	48	7^a^	38	5^a^
Vegan	23	48	-	-
Vegetarian + fish	10	21	1	3
Vegetarian, no fish	6	13	1	3
Omnivore	8	17	31	82
Don’t know/don’t want to answer	1	2	5	13
*Children aged 1–2 years*				
Number of Children	47	7^a^	65	8^a^
Vegan	17	36	-	-
Vegetarian + fish	15	32	-	-
Vegetarian, no fish	7	15	2	3
Omnivore	7	15	63	97
Don’t know/don’t want to answer	1	2	-	-
*Children aged 3–6 years*				
Number of Children	50	7^a^	87	11^a^
Vegan	16	32	-	-
Vegetarian + fish	11	22	2	2
Vegetarian, no fish	7	14	-	-
Omnivore	16	32	84	97
Don’t know/don’t want to answer	-	-	1	1
*Children aged 7–10 years*				
Number of Children	56	8^a^	74	9^a^
Vegan	9	16	-	-
Vegetarian + fish	18	32	1	1
Vegetarian, no fish	7	13	1	1
Omnivore	22	39	69	93
Don’t know/don’t want to answer	-	-	3	4
*Children aged 11–13 years*				
Number of Children	52	7^a^	59	7^a^
Vegan	12	23	1	2
Vegetarian + fish	14	27	1	2
Vegetarian, no fish	10	19	-	-
Omnivore	15	29	57	97
Don’t know/don’t want to answer	1	2	-	-
*Children aged 14–17 years*				
Number of Children	63	9^a^	63	8^a^
Vegan	20	32	-	-
Vegetarian + fish	16	25	1	2
Vegetarian, no fish	7	11	2	3
Omnivore	19	30	59	94
Don’t know/don’t want to answer	1	2	1	2

For children within each age category, the table displays the number of children reported by respondents to follow each type of diet (vegan; vegetarian + fish; vegetarian, no fish; omnivore)

^a^This percentage refers to the respective total n of vegan/omnivorous participants. The percentages below refer to the respective number of children in each age category

Female vegan respondents who indicated that they had been following a vegan diet for 10 years or more and indicated that they had been pregnant in the past 10 years (*n* = 6) were asked about their diets during pregnancy, indicating to have followed a vegan (*n* = 4) and vegetarian without fish (*n* = 2) diet.

### Nutritional knowledge and information

#### Information sources

Individuals who reported actively keeping informed on nutrition (86% of the vegan sample and 64% of the omnivorous sample) were asked to list sources of information they used. Responses to this open question were grouped (see Table 2 for frequencies), with the internet in general being named most frequently by vegan and omnivorous participants alike.

**Table 2:** Frequency of information sources named by respondents self-reporting to keep actively informed on nutrition

	Vegan Participants (*n* = 636)		Omnivorous Participants (*n* = 525)	
	n	%	n	%
Internet (general)	408	64	377	72
Online social networks	125	20	69	13
Newspapers and magazines	85	13	93	18
Books	126	20	37	7
Google	46	7	57	11
Websites on nutrition and health	57	9	34	6
Television	34	5	52	10
Other sources on the internet	44	7	25	5
Friends, acquaintances or family	29	5	32	6
Other media	25	4	17	3
Documentaries	31	5	3	1
Organizations	31	5	2	0
Online-forums	9	1	15	3
Medical professionals	11	2	10	2
Nutritionists	5	1	15	3
Informative meetings	15	2	4	1
Scientific studies	13	2	5	1
Other	38	6	30	6
Do not know/do not want to answer	150	24	87	17

Response categories were derived based on the most frequent answers. Multiple responses were possible

Individuals who reported actively keeping informed on nutrition rated the degree to which specific sources of information were able to offer helpful information regarding nutrition (see Table 3). Books, scientific studies, and sources on the internet other than online forums or social networks received the highest ratings among both vegan and omnivorous respondents.

**Table 3:** Means, standard deviations and group comparisons (vegan/omnivorous participants’ responses) for the degree to which participants agreed each source of information was able to offer helpful information regarding nutrition

	Vegan Participants (*n* = 636)			Omnivorous Participants (*n* = 525)			Group comparison				
	M	SD	n^a^	M	SD	n^b^	df_1_	df_2_	*F*	*p*	*ɳ* ^*2*^
Online-forums	3.45	1.27	626	3.57	1.20	511	1	1001	1.271	0.260	0.001
Online social networks	3.57	1.23	626	3.45	1.24	508	1	1001	2.430	0.119	0.002
Other sources on the internet	3.91	1.10	627	4.00	0.92	510	1	1001	1.433	0.232	0.001
Magazines	3.35	1.23	624	3.52	1.13	512	1	1001	5.348	0.021	0.005
Books	3.97	1.10	624	3.72	1.18	508	1	1001	13.182	0.000	0.013
Friends/acquaintances	3.39	1.17	620	3.63	1.05	509	1	1001	12.361	0.000	0.012
Family	2.99	1.34	624	3.68	1.08	509	1	1001	80.807	0.000	0.075
Scientific studies, e.g. by the German Nutrition Society	3.68	1.23	619	3.86	1.17	505	1	1001	4.418	0.036	0.004
Physicians	3.14	1.37	620	3.77	1.18	506	1	1001	53.062	0.000	0.050
Nutritionists	3.27	1.44	595	3.52	1.40	494	1	1001	8.227	0.004	0.008

Higher scores indicate greater agreement (1 = strongly disagree; 5 = strongly agree)

^a^The number of respondents who did not know/did not want to answer ranged between n = 9–41

^b^The number of respondents who did not know/did not want to answer ranged between n = 13–31

To further investigate if people who follow a vegan diet and people who follow an omnivorous diet differ in their ratings regarding the helpfulness of different information sources overall, a multivariate analysis of variance (MANOVA) was conducted.

A one-way MANOVA showed a statistically significant difference between the two groups on their ratings of information sources (F(10, 992) = 16.820, *p* <.001, partial η^2^ = 0.145, Pillai’s Trace = 0.145). Post-hoc univariate ANOVAs were conducted for every dependent variable. Vegan and omnivorous participants differed significantly in their ratings of magazines, books, friends/acquaintances, family, scientific studies, physicians, and nutritionists (see Table 3). Vegan participants rated books significantly more highly for offering helpful information regarding nutrition than omnivorous participants. Omnivorous participants find magazines, friends/acquaintances, family, scientific studies, physicians, and nutritionists in this regard significantly more helpful than vegan participants.

#### Advantages and disadvantages

**Fig. 1:**
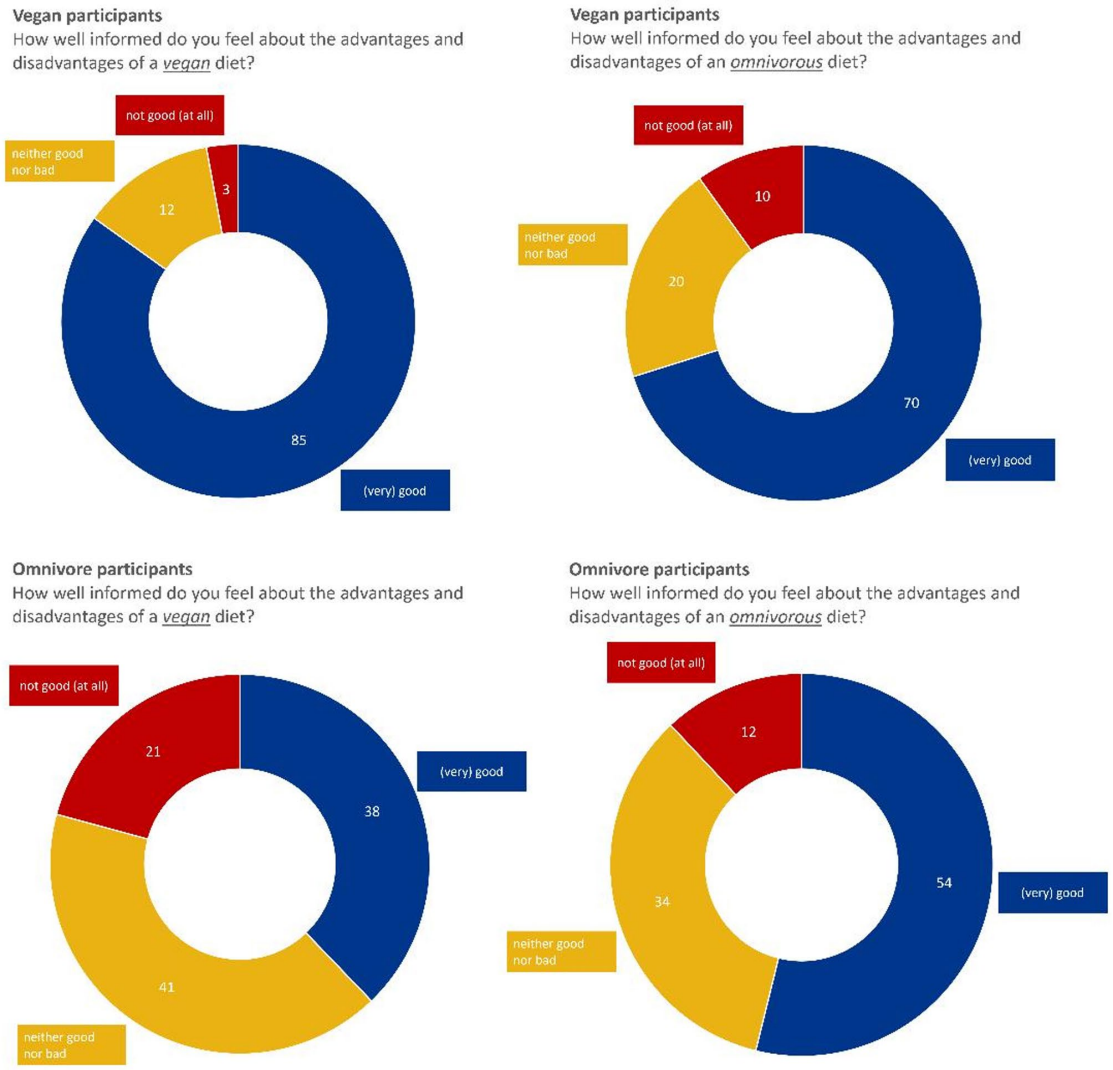
Informedness about advantages and disadvantages of vegan and omnivore diets

Regardless of their current dietary habits, participants were asked how well informed they felt about the advantages and disadvantages of vegan and omnivorous diets (1 = very good; 5 = not good at all). People who follow a vegan diet felt significantly better informed about the vegan than the omnivorous diet (t(713) = −10.598; *p* <.001; d = 1.577) whereas the omnivorous group felt significantly better informed about the omnivorous diet (see Fig. 1 for descriptive results; t(773) = 9.782; *p* <.001; d = 4.256).

Furthermore, the vegan group felt significantly better informed about either nutritional style (see Table 4). This effect was strong for the vegan diet (d = 1.270) and moderate for the omnivorous diet (d = 0.426).

**Table 4:** Means, standard deviations and group comparisons (vegans/omnivorous participants’ responses) for the degree to which participants felt informed about the advantages/disadvantages of a vegan and omnivore diet respectively

	Vegan Participants (*n* = 738)			Omnivorous Participants (*n* = 824)			Group comparison		
	M	SD	n^a^	M	SD	n^b^	t (degrees of freedom)	p	Cohen’s d
Vegan Diet	1.65	0.82	729	2.81	1.02	780	t(1471.69)= −24.361	< 0.001	1.270
Omnivorous Diet	2.05	1.07	717	2.47	0.96	793	t(1444.26)= −8.104	< 0.001	0.426

Lower scores indicate better informedness (1 = very good; 5 = not good at all)

^a^The number of respondents who did not know/did not want to answer ranged between n = 9–21

^b^The number of respondents who did not know/did not want to answer ranged between n = 31–44

Among individuals reporting to see advantages to their current dietary habits (88% of the vegan sample, *n* = 653; 58% of the omnivorous sample, *n* = 476), vegan participants most frequently listed health in general (35%), animal protection (29%), positive effects on environment or climate (25%), positive effects on sleep/energy/productivity (15%), positive effects on weight or looks, e.g. skin (12%), positive effects on physical health (11%), an alignment of the dietary habit with moral convictions/conscience (11%), positive effects on wellbeing/mental health (8%), a reduced risk of developing chronic/future diseases (6%), resilience against acute illnesses/a strengthened immune system (5%), taste/enjoyment of cooking and eating (5%), a balanced/varied diet (4%) or other advantages (8%). 22% of vegan respondents did not know/did not want to answer. Omnivorous participants listed a balanced/varied diet (33%), the consumption of all necessary nutrients/no nutrient deficiencies (28%), health in general (15%), taste/enjoyment of cooking and eating (8%), positive effects on weight or looks, e.g. skin (6%), positive effects on sleep/energy/productivity (6%), positive effects on well-being or mental health (3%) or other advantages (1%), with 18% of the omnivorous respondents indicating they did not know/did not want to answer.

Among individuals who reported seeing disadvantages to their current dietary habits (39% of the vegan sample, *n* = 290; 22% of the omnivore sample, *n* = 178), difficulties in the practical implementation (e.g. product availability, 21%), the necessity to supplement nutrients/pay close attention to nutrient intake (15%), social pressure/discussions/conflicts in the social environment (13%), nutrient deficiency (11%), high costs (9%), reduced enjoyment of food (4%) and negative effects on health (3%) were most frequently listed by vegan participants, with 3% listing other disadvantages and 38% indicating they did not know/did not want to answer. Omnivorous participants listed an unbalanced diet (33%), negative effects on health (22%), too much fat/carbohydrates/sugar/calories (18%), negative effects on environment/climate (13%), negative effects on animals (10%), too much meat/too little fruit and vegetable intake (10%), negative effects on weight (6%), nutrient deficiency (4%), difficulties in practical implementation (3%), high costs (3%), pleasure reduction (3%) and challenges in terms of self-control (3%) as disadvantages, with 1% of the respondents listing other disadvantages and 17% indicating they did not know/did not want to answer.

Respondents were asked to rate their agreement with different statements regarding their current diet’s impact on their health (see Table 5).

**Table 5:** Means (M), standard deviations (SD) and group comparisons for respondents’ agreement with statements on their current diet’s impact on their health

	Vegan Participants (*n* = 738)			Omnivorous Participants (*n* = 824)			Group comparison				
	M	SD	n^a^	M	SD	n^b^	df_1_	df_2_	*F*	*p*	*ɳ* ^*2*^
(1) The probability of developing diabetes increases.	2.26	1.49	699	2.65	1.22	729	1	1195	26.334	0.000	0.022
(2) The probability of developing a cardiovascular problem increases.	2.29	1.49	709	2.65	1.16	724	1	1195	21.020	0.000	0.017
(3) Metabolism is impaired.	2.16	1.37	699	2.53	1.16	722	1	1195	26.272	0.000	0.022
(4) Oxygen transport is impaired.	2.19	1.42	676	2.46	1.14	697	1	1195	17.094	0.000	0.014
(5) Cholesterol levels are reduced.	4.04	1.14	673	3.06	1.11	701	1	1195	226.204	0.000	0.159
(6) The probability of developing cancer is reduced.	3.95	1.18	688	2.97	1.11	665	1	1195	208.986	0.000	0.149
(7) The vitamin and mineral supply is improved.	3.82	1.13	701	3.60	1.04	736	1	1195	22.288	0.000	0.018
(8) It becomes easier to reach or maintain a healthy body weight.	4.00	1.09	702	3.40	1.12	754	1	1195	109.285	0.000	0.084

Higher scores indicate higher agreement (1 = strongly disagree; 5 = strongly agree)

^a^The number of respondents who did not know/did not want to answer ranged between n = 29–65

^b^The number of respondents who did not know/did not want to answer ranged between n = 70–159

A one-way MANOVA showed a significant difference between the two groups on their overall agreement across all statements on the influence of the current diet on health, F(8, 1188) = 3604.207, *p* <.001, partial η^2^ = 0.960, Pillai’s Trace = 0.960. Post-hoc univariate ANOVAs for all dependent variables (see Table 5) showed a significant difference between vegan and omnivorous participants’ ratings on every statement. Compared to vegan participants, omnivorous participants considered their diets more likely to increase their chances of developing diabetes (1) or cardiovascular problems (2), result in impaired metabolism (3) and oxygen transport (4). Vegan participants considered their diet more likely to reduce Cholesterol levels (5), decrease the risk of cancer (6), improve their supply of vitamins and minerals (7), and make it easier to achieve or maintain body weight goals (8) than did omnivorous participants.

Vegan participants who reported that switching to a vegan diet impacted their health and well-being (70% of the whole vegan sample, *n* = 519) were asked to list the specific impacts their change of dietary habit had. Positive impacts in general (26%), increased energy/productivity/fitness (22%), improved overall wellbeing/mental health (14%), positive impact on weight (12%), reduced health complaints (11%), improved skin/hair/nails (10%), improved digestion (10%), improved sleep/tiredness levels (6%), resilience against acute illnesses (e.g. infections)/improved immune system (6%), improved wellbeing following meals (5%), and a better conscience (4%) were most frequently listed, with 28% of respondents indicating they did not know/did not want to answer.

#### Deficiency risks

Respondents who saw a risk of vitamin or mineral deficiency in their current diet without the regular use of supplements (57% of the vegan sample and 17% of the omnivorous sample) were asked which vitamin or mineral posed deficiency risks (closed-ended; see Table 6 for results). Among both groups, iron, calcium and vitamin D were named frequently, but while the vegan respondents most frequently indicated vitamin B12 as a possible deficiency risk, the omnivorous group most frequently indicated magnesium.

**Table 6:** Frequency of vitamins/minerals at risk of deficiency as perceived by the respondents

	Vegan Participants (*n* = 419)		Omnivorous Participants (*n* = 140)	
	n	%	n	%
Vitamin B12/cobalamin	271	65	40	29
Iron	128	31	57	41
Calcium	101	24	43	31
Vitamin D	88	21	48	34
Magnesium	61	15	60	43
Iodine	52	12	18	13
Vitamin B1	49	12	24	17
Zinc	42	10	31	22
Vitamin B2	41	10	25	18
Vitamin A	45	11	20	14
Vitamin B6	37	9	19	14
Vitamin C	33	8	28	20
Vitamin B9/folic acid	31	7	21	15
Phosphorus	30	7	15	11
Vitamin K	25	6	13	9
Vitamin E	21	5	15	11
Vitamin H/biotin	21	5	16	11
Don’t know/prefer not to answer	5	1	15	11

Multiple responses were possible

**Fig. 2:**
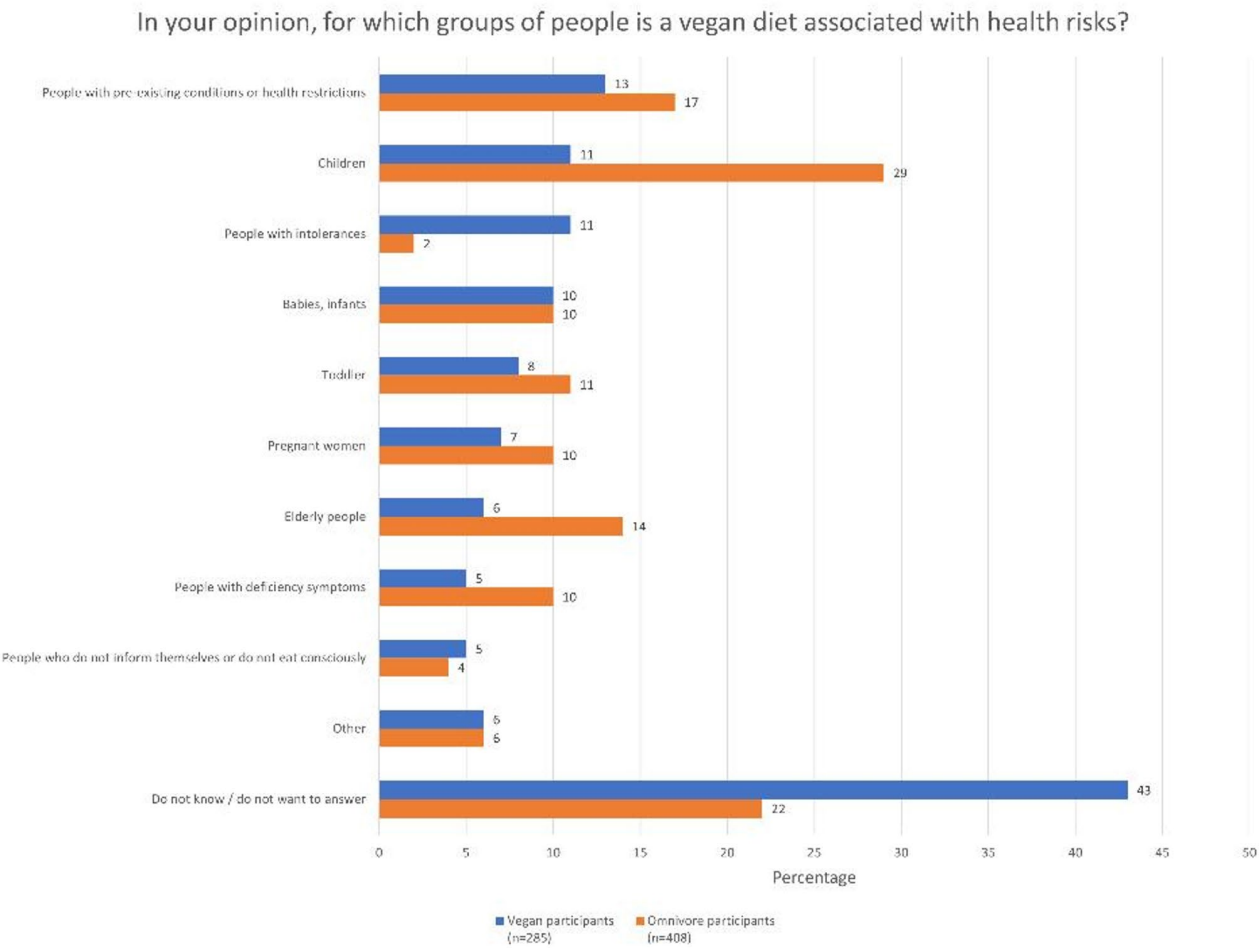
Frequencies of groups of people who are at risk when adopting a vegan diet as perceived by the respondents. Response categories were derived based on the most frequent answers. Multiple responses were possible

Individuals who reported to believe that there are groups of people for whom a vegan diet is associated with health risks (39% of the vegan sample and 50% of the omnivorous sample) were asked to name the corresponding groups of people (open-ended). Vegan and omnivorous respondents most frequently listed children and people with pre-existing conditions or health restrictions as vulnerable groups (see Fig. 2).

Respondents were then presented with different potentially vulnerable groups (closed-ended) and asked to indicate their agreement with statements about these groups being at risk when adopting a vegan diet (see Table 7). People who follow an omnivorous diet on average agreed more strongly across all statements, indicating they perceived the risks of a vegan diet greater across all listed population groups compared to vegan respondents.

**Table 7:** Means, standard deviations and group comparisons (vegan/omnivorous participants’ responses) for the degree to which participants felt different groups of people at risk when adopting a vegan diet

	Vegan Participants (*n* = 285)			Omnivorous Participants (*n* = 408)			Group comparison				
Risk Group	M	SD	n^a^	M	SD	n^b^	df_1_	df_2_	*F*	*p*	*ɳ* ^*2*^
Babies (< 12 months)	2.98	1.51	658	4.08	1.23	718	1	1214	207.934	0.000	0.146
Infants (1–3 years of age)	2.88	1.48	674	4.08	1.15	725	1	1214	273.267	0.000	0.184
Children (4–12 years of age)	2.74	1.47	684	3.93	1.17	726	1	1214	260.517	0.000	0.177
Adolescents (13–18 years of age)	2.39	1.44	692	3.44	1.18	721	1	1214	184.899	0.000	0.132
Women during pregnancy	2.76	1.46	676	3.92	1.14	721	1	1214	270.149	0.000	0.182
Women who breastfeed	2.64	1.46	667	3.88	1.16	704	1	1214	286.301	0.000	0.191
Older adults aged 65 or over	2.37	1.45	685	3.60	1.19	707	1	1214	266.469	0.000	0.180
Persons with chronic illnesses	2.62	1.43	679	3.77	1.14	700	1	1214	263.418	0.000	0.178
Adults (19–64 years of age)	2.22	1.47	698	2.86	1.16	717	1	1214	76.394	0.000	0.059

Higher scores indicate higher agreement (1 = strongly disagree; 5 = strongly agree)

^a^The number of respondents who did not know/did not want to answer ranged between n = 40–80

^b^The number of respondents who did not know/did not want to answer ranged between n = 98–124

A one-way MANOVA showed that this overall difference in the perception between the vegan and the omnivorous participants was significant, F(9, 1206) = 42.940, *p* <.001, partial η^2^ = 0.243, Pillai’s Trace = 0.243. Post-hoc univariate ANOVAs were conducted for every dependent variable (see Table 7). Vegan and omnivorous participants’ agreement ratings differed significantly for every single statement, indicating that omnivorous respondents perceived every single one of the listed groups to be at more risk when adopting a vegan diet than did vegan respondents.

Respondents who agreed with the statement of any groups being at risk when adopting a vegan diet were given a list of different measures that might minimize such risks and asked to indicate whether they would expect these measures to minimize any potential health risks relating to a vegan diet effectively (see Table 8). Vegan as well as omnivorous respondents most frequently indicated regular medical check-ups/consultations, the consumption of nutritional supplements, and the consumption of fortified foodstuffs as measures expected to minimize health risks across all potential risk groups.

**Table 8:** Frequencies of measures, intended to minimize the risks of adopting a vegan diet for different groups of people

	Vegan Participants		Omnivorous Participants	
	n	%	n	%
Babies (< 12 months)	*n* = 397		*n* = 624	
Regular medical check-ups/consultations	242	61	368	59
Consumption of nutritional supplements	160	40	190	30
Consuming fortified foodstuffs	183	46	223	36
By seeking nutrition consultation	105	26	168	27
Other	26	7	24	4
None of the above	21	5	88	14
Don’t know/prefer not to answer	12	3	58	9
Infants (1–3 years of age)	*n* = 382		*n* = 648	
Regular medical check-ups/consultations	212	55	377	58
Consumption of nutritional supplements	168	44	194	30
Consuming fortified foodstuffs	181	47	240	37
By seeking nutrition consultation	99	26	168	26
Other	14	4	22	3
None of the above	20	5	96	15
Don’t know/prefer not to answer	17	4	56	9
Children (4–12 years of age)	*n* = 366		*n* = 637	
Regular medical check-ups/consultations	193	53	361	57
Consumption of nutritional supplements	165	45	215	34
Consuming fortified foodstuffs	176	48	243	38
By seeking nutrition consultation	99	27	177	28
Other	10	3	15	2
None of the above	18	5	93	15
Don’t know/prefer not to answer	13	4	54	8
Adolescents (13–18 years of age)	*n* = 295		*n* = 569	
Regular medical check-ups/consultations	138	47	302	53
Consumption of nutritional supplements	127	43	208	37
Consuming fortified foodstuffs	123	42	235	41
By seeking nutrition consultation	64	22	161	28
Other	7	2	16	3
None of the above	14	5	69	12
Don’t know/prefer not to answer	11	4	56	10
Women during pregnancy	*n* = 364		*n* = 637	
Regular medical check-ups/consultations	201	55	358	56
Consumption of nutritional supplements	164	45	235	37
Consuming fortified foodstuffs	159	44	217	34
By seeking nutrition consultation	117	32	202	32
Other	8	2	14	2
None of the above	15	4	95	15
Don’t know/prefer not to answer	13	4	61	10
Women who breastfeed	*n* = 347		*n* = 614	
Regular medical check-ups/consultations	195	56	339	55
Consumption of nutritional supplements	150	43	223	36
Consuming fortified foodstuffs	145	42	221	36
By seeking nutrition consultation	94	27	195	32
Other	7	2	15	2
None of the above	21	6	88	14
Don’t know/prefer not to answer	14	4	57	9
Older adults aged 65 or over	*n* = 280		*n* = 584	
Regular medical check-ups/consultations	136	49	358	61
Consumption of nutritional supplements	135	48	266	46
Consuming fortified foodstuffs	118	42	226	39
By seeking nutrition consultation	73	26	180	31
Other	5	2	9	2
None of the above	10	4	66	11
Don’t know/prefer not to answer	10	4	50	9
Persons with chronic illnesses	*n* = 349		*n* = 613	
Regular medical check-ups/consultations	197	56	371	61
Consumption of nutritional supplements	170	49	230	38
Consuming fortified foodstuffs	137	39	207	34
By seeking nutrition consultation	111	32	191	31
Other	8	2	12	2
None of the above	16	5	83	14
Don’t know/prefer not to answer	15	4	59	10
Adults (19–64 years of age)	*n* = 254		*n* = 441	
Regular medical check-ups/consultations	113	44	224	51
Consumption of nutritional supplements	100	39	179	41
Consuming fortified foodstuffs	95	37	161	37
By seeking nutrition consultation	57	22	130	29
Other	2	1	8	2
None of the above	14	6	53	12
Don’t know/prefer not to answer	6	2	47	11

### Nutritional habits and consumption behaviors

When prompted regarding their nutritional habits before adopting a vegan diet, 322 vegan respondents indicated having followed a vegetarian diet (44%), 226 reported they had followed a vegetarian diet but consumed fish (31%), 173 indicated they had followed an omnivorous diet (23%), and 17 did not know/did not want to answer (2%). When asked how long they had followed a vegan diet, 123 respondents indicated it had been less than a year (17%), 143 reported having followed a vegan diet for 1–2 years (19%), 2–3 years (*n* = 143, 19%), 3–4 years (*n* = 74, 10%), 4–5 years (*n* = 73, 10%), 5–10 years (*n* = 105, 14%), 10–15 years (*n* = 36, 5%) or longer (*n* = 30, 4%), with 11 participants indicating they did not know/did not want to answer (1%).

Respondents were asked how frequently they ate ready meals, processed foods, and home-cooked meals. 20% of the vegan sample as opposed to 9% of the omnivorous sample reported never eating ready meals (see Table 9). Eating self-prepared foods on a daily basis was reported by 52% of the vegan sample as opposed to 38% of the omnivorous sample.

**Table 9:** Self-reported consumption frequencies of ready meals, processed foods and self-prepared foods

	Vegan Paticipants (*n* = 738)						Omnivorous Participants (*n* = 824)					
Frequency of consumption	Ready meals		Processed foods		Self-prepared foods		Ready meals		Processed foods		Self-prepared foods	
	n	%	n	%	n	%	n	%	n	%	n	%
Never	149	20	57	8	19	3	73	9	21	3	7	1
Less than once a month	171	23	99	13	22	3	166	20	78	9	18	2
1–3 times per month	158	21	124	17	53	7	198	24	107	13	28	3
Once a week	122	17	185	25	65	9	216	26	217	26	70	8
Several times a week	73	10	172	23	183	25	124	15	263	32	352	43
Daily	51	7	78	11	385	52	13	2	69	8	317	38
Don’t know/prefer not to answer	14	2	23	3	11	1	34	4	69	8	32	4

Respondents were asked how often they ate vegetables, salad, and/or fruit. While 49% of vegan respondents reported to consume vegetables, salad, and/or fruit 3 times or more per day, only 17% of omnivorous respondents did so (see Table 10).

**Table 10:** Self-reported consumption frequencies of fruit, salad and/or vegetables

	Vegan Participants (*n* = 738)		Omnivorous Participants (*n* = 824)	
Frequency of consumption: portions per day	Fruit, salad and/or vegetables		Fruit, salad and/or vegetables	
	n	%	n	%
Never	16	2	6	1
Less than 1 per week	34	5	41	5
Less than 1 per day	71	10	201	24
1–2 per day	246	33	409	50
3–4 per day	235	32	111	13
5 or more per day	127	17	32	4
Don’t know/prefer not to answer	9	1	24	3

Asked about their meat consumption, 8% of omnivorous respondents (*n* = 66) reported consuming less than one portion per week. 27% of the respondents (*n* = 221) reported consuming 1–2 portions, 38% (*n* = 314) 2–4 portions, 14% (*n* = 118) 4–6 portions, and 6% (*n* = 52) 4–6 portions per week, using a portion of 150 g meat as a reference. Some respondents did not know/preferred not to answer (6%, *n* = 53).

Respondents reporting to take nutritional supplements (69% of the vegan sample, *n* = 511 and 28% of the omnivore sample, *n* = 227) were queried whether a chronic illness was the reason for taking supplements. 34% of vegan (*n* = 174) and 20% (*n* = 46) of the omnivorous participants answered in the affirmative.

After being asked to list any supplements, they were taking, respondents were queried regarding consumption frequency. Many supplements were taken daily (see Table 11), with zinc (54%) and Vitamin K (54%) being taken most frequently by people who follow a vegan diet. The supplements being taken most frequently on a daily basis among the omnivorous respondents were Vitamin H (Biotin, 70%) and Vitamin B9 (folic acid, 67%).

**Table 11:** Frequencies of supplement use

	Vegan Participants		Omnivorous Participants	
	n	%	n	%
Vitamin B12 (Cobalamin)	*n* = 335		*n* = 78	
Less frequently than once a month	14	4	2	3
About 1–3 times a month	18	5	8	10
About once a week	47	14	10	13
Several times a week	90	27	16	21
Daily	164	49	41	53
Don’t know/prefer not to answer	2	1	1	1
Vitamin D	*n* = 217		*n* = 98	
Less frequently than once a month	7	3	1	1
About 1–3 times a month	13	6	10	10
About once a week	43	20	10	10
Several times a week	38	18	28	29
Daily	112	52	47	48
Don’t know/prefer not to answer	4	2	2	2
Magnesium	*n* = 155		*n* = 127	
Less frequently than once a month	6	4	5	4
About 1–3 times a month	18	12	17	13
About once a week	24	15	16	13
Several times a week	40	26	38	30
Daily	67	43	47	37
Don’t know/prefer not to answer	-	-	4	3
Calcium	*n* = 115		*n* = 59	
Less frequently than once a month	9	8	2	3
About 1–3 times a month	29	25	7	12
About once a week	16	14	12	20
Several times a week	20	17	12	20
Daily	40	35	24	41
Don’t know/prefer not to answer	1	1	2	3
Iron	*n* = 104		*n* = 80	
Less frequently than once a month	7	7	2	3
About 1–3 times a month	19	18	7	9
About once a week	17	16	17	21
Several times a week	20	19	17	21
Daily	41	39	32	40
Don’t know/prefer not to answer	*-*	-	*5*	6
Vitamin B1	*n* = 85		*n* = 40	
Less frequently than once a month	8	9	1	3
About 1–3 times a month	6	7	4	10
About once a week	14	16	5	13
Several times a week	18	21	8	20
Daily	38	45	21	53
Don’t know/prefer not to answer	1	1	1	3
Zinc	*n* = 83		*n* = 63	
Less frequently than once a month	2	2	-	-
About 1–3 times a month	5	6	2	3
About once a week	7	8	4	6
Several times a week	22	27	14	22
Daily	45	54	40	63
Don’t know/prefer not to answer	2	2	3	5
Vitamin B6	*n* = 76		*n* = 38	
Less frequently than once a month	4	5	-	-
About 1–3 times a month	7	9	7	18
About once a week	12	16	2	5
Several times a week	19	25	6	16
Daily	33	43	21	55
Don’t know/prefer not to answer	1	1	2	5
Vitamin B9 (folic acid)	*n* = 67		*n* = 33	
Less frequently than once a month	5	7	-	-
About 1–3 times a month	5	7	2	6
About once a week	10	15	2	6
Several times a week	14	21	6	18
Daily	32	48	22	67
Don’t know/prefer not to answer	*1*	1	*1*	3
Vitamin C	*n* = 76		*n* = 79	
Less frequently than once a month	1	1	-	-
About 1–3 times a month	4	5	7	9
About once a week	6	8	11	14
Several times a week	22	29	21	27
Daily	40	53	37	47
Don’t know/prefer not to answer	3	4	3	4
Vitamin B2	*n* = 64		*n* = 32	
Less frequently than once a month	3	5	-	-
About 1–3 times a month	7	11	2	6
About once a week	6	9	3	9
Several times a week	17	27	7	22
Daily	31	48	19	59
Don’t know/prefer not to answer	-	-	1	3
Vitamin K	*n* = 65		*n* = 19	
Less frequently than once a month	2	3	-	-
About 1–3 times a month	1	2	-	-
About once a week	11	17	1	5
Several times a week	13	20	4	21
Daily	35	54	12	63
Don’t know/prefer not to answer	3	5	2	11
Iodine	*n* = 67		*n* = 27	
Less frequently than once a month	6	9	1	4
About 1–3 times a month	10	15	5	19
About once a week	12	18	2	7
Several times a week	16	24	5	19
Daily	23	34	13	48
Don’t know/prefer not to answer	-	-	1	4
Vitamin A	*n* = 59		*n* = 31	
Less frequently than once a month	7	12	1	3
About 1–3 times a month	5	8	3	10
About once a week	10	17	4	13
Several times a week	14	24	7	23
Daily	23	39	15	48
Don’t know/prefer not to answer	-	-	1	3
Vitamin H (Biotin)	*n* = 39		*n* = 20	
Less frequently than once a month	2	5	-	-
About 1–3 times a month	2	5	-	-
About once a week	7	18	2	10
Several times a week	10	26	3	15
Daily	18	46	14	70
Don’t know/prefer not to answer	-	-	1	5
Phosphorus	*n* = 34		*n* = 18	
Less frequently than once a month	3	9	-	-
About 1–3 times a month	8	24	3	17
About once a week	5	15	2	11
Several times a week	6	18	6	33
Daily	11	32	6	33
Don’t know/prefer not to answer	1	3	1	6

### Health-related behaviors

Respondents were asked to describe their health on a scale from 1 (very poor) to 5 (very good). Vegan and omnivorous participants differed significantly (Vegan: M = 3.94, SD = 1.10; Omnivore: M = 3.82, SD = 1.00; t(1431.61) = 2.27, *p* =.024, d = 0.12). Although the effect size was small, vegan respondents described themselves as healthier than omnivorous respondents.

To assess the physical activity levels, an IPAQ-score was calculated according to the International Physical Activity Questionnaire guidelines [29]. Of the 563 vegan respondents, 31% were categorized as highly physically active, 27% as moderately physically active, and 41% as little physically active. Of the 658 omnivorous respondents, 29% were highly, 29% moderately, and 42% little physically active. There were no significant differences in self-reported physical activity between vegan and omnivorous respondents (U = 181207.00, Z = − 0.699, *p* =.485).

Participants were asked about their consumption of alcoholic beverages (see Table 12). More vegan than omnivorous respondents reported not to consume alcohol at all and more omnivorous respondents reported drinking more frequently than vegan respondents. However, of the vegan participants who did consume alcohol regularly, more seemed to drink larger amount more frequently (daily consumption of 5 or 6 units and more frequent consumption of 6 or more units) than omnivorous participants who regularly consumed alcohol. This could indicate a more heterogenous pattern of drinking behavior in the vegan than the omnivorous sample, with more individuals choosing complete abstinence but also more drinking large amounts more frequently than in the omnivorous sample, which tended more towards more regular consumption of small or moderate amounts.

**Table 12:** Frequencies of alcohol consumption

	Vegan Participants		Omnivorous Participants	
	n	%	n	%
Frequency of consumption	*n* = 738		*n* = 824	
Never	232	31	185	22
About once per month	179	24	188	23
2–4 times per month	157	21	227	28
2–3 times per week	111	15	127	15
4 times per week	39	5	60	7
Don’t know/prefer not to say	20	3	37	4
Daily consumption	*n* = 468		*n* = 602	
1 or 2 units	231	48	319	53
3 or 4 units	128	26	167	28
5 or 6 units	83	17	61	10
7 or 8 units	20	4	13	2
9 or more units	13	3	17	3
Don’t know/prefer not to say	11	2	25	4
Consumption of 6 units or more	*n* = 468		*n* = 602	
Never	113	23	171	28
Less than once per month	154	32	189	31
Once per month	123	25	120	20
Once per week	63	13	80	13
Daily or almost daily	22	5	21	3
Don’t know/prefer not to say	11	2	21	3

#### Smoking behaviors

Participants were asked about their smoking behaviors (see Table 13). A chi-square test was used to compare vegan and omnivorous participants’ smoking status. No expected cell frequencies were below 5. Results show no significant correlation between dietary group and smoking status, χ² [2] = 11.04, *p* =.004, φ = 0.86, indicating that smoking status didn’t differ between the groups.

**Table 13:** Smoking status

	Vegan Participants (*n* = 738)		Omnivorous Participants (*n* = 824)	
	n	%	n	%
Smoking				
Yes	170	23	233	28
No, I used to smoke, but not now	206	28	176	21
No, I’ve never smoked regularly	339	46	386	47
Don’t know/prefer not to say	23	3	29	4

Participants who reported to currently smoke were asked how many cigarettes they smoke per day. Vegan smokers reported to consume on average 10.19 (SD = 8.562) cigarettes per day and omnivorous smokers reported to consume on average 14.49 (SD = 8.182) cigarettes per day. There was a significant difference in cigarette consumption between the two groups (t(305.67) = −4.812, p = < 0.001), indicating that omnivorous participants who smoke consumed more cigarettes per day than vegan participants who smoke.

## Discussion

### Motivation and key triggers for diet adoption

Motivations for adopting a vegan diet align with previous robust findings [30–33]; ethical, health, and ecological reasons were the most influential. Nonetheless, North et al. [31] observed similar motivations for dietary choices across vegan, vegetarian, and omnivorous groups. Specifically, “health” was a factor frequently cited by all three groups in their study. Yet, a difference was observed in the interpretation of this health motivator: Vegans and vegetarians primarily emphasized personal health benefits, whereas omnivores stressed the nutritional aspect of their diet. Since health could be a key motivator for all dietary styles, it seems vital to translate scientific evidence on the health benefits of a vegan diet and how they contrast with the benefits of other dietary styles into easily accessible nutrition guidelines and behavior change interventions.

Approximately two-thirds of vegans were prompted by a key experience that triggered their dietary change. Documentaries on animal rights, husbandry, or veganism were a frequent trigger, often consumed in video format rather than via radio or text. The prevalence of video content as a primary trigger suggests that social media could play a critical role in prompting dietary considerations, as it frequently utilizes video formats and facilitates the sharing of content that elicits strong reactions.

This hypothesis is supported by previous research linking digital exposure to behavioral intent: Kadel et al. [34] found that exposure to vegan content on Instagram correlated with increased vegan eating intentions via psychological mechanisms like attitude and self-identity. This aligns with the TPB [26], which posits that attitudes strongly influence intentions, a key predictor of behavior [27]. Consequently, utilizing social media as an information and dissemination channel could enhance the success of public health interventions promoting a vegan diet.

### Social environment and network influence

Social influence on adopting a vegan diet showed heterogeneous patterns, arising from both close and distant relationships. Since distant influence largely came from influencers and YouTubers, social media emerges as a relevant factor in opinion formation.

The social component of adoption is supported by the finding that the majority of vegan participants had other vegans in their networks (fewer than 20% had none). This observation suggests two contrasting elements: while the motivation to adopt and maintain the diet seems to have a social dimension for most individuals, the diet can still be adopted or maintained independently of immediate social influences. This aligns with existing research, such as a systematic review by Chung et al. [35], showing that adolescent diet is significantly associated with peer behavior.

Furthermore, D’Souza, Brouwer, and Singaraju [36] confirmed a significant relationship between social norms and the behavioral intention to maintain a vegan diet (despite social norms being the weakest predictor compared to attitudes and perceived behavioral control, PBC). In a related finding, Williams et al. [37] demonstrated that social acceptance from family and friends is important for diet maintenance. These results indicate the need to consider social aspects—for instance, the target group of the intervention—when developing public health strategies aimed at promoting vegan diet. This is directly supported by the TPB [26], where these social influences – including peer behavior and acceptance from family and friends – form the basis of subjective norms, a key predictor of behavioral intention [36]. The need for social acceptance and appropriate support extends to the clinical context, where a lack of flexibility and acknowledgement of vegan values in areas like eating disorder treatment can present a significant social and clinical challenge [38].

### Diet of children in vegan and omnivorous households

This study revealed that children in omnivorous households predominantly followed omnivorous diets across all age ranges. The distribution was markedly heterogeneous for vegan households; for example, among children aged one to two years, only 15% were omnivorous, while 36% followed a vegan diet, and the remainder were vegetarian (with or without fish). To date, scientific literature contains limited data on whether parental dietary choice (vegan or omnivorous) is applied to their children. Consequently, the findings from present study are relevant for the development of behavioral interventions. They demonstrate that children might be implicitly reached as a secondary audience – a finding that requires systematic consideration alongside the primary target groups.

### Differences in perceived risks

The findings regarding parental feeding practices align with our results on risk perceptions regarding a vegan diet.

Omnivorous respondents consistently expressed greater perceived risk for almost all groups examined, including both vulnerable populations and adults aged 19–64. Risk perception was highest for women who breastfeed, infants aged 1–3 years, pregnant women, and older adults (65+), and lowest for adults aged 19–64.

This indicates that omnivorous participants generally considered a vegan diet more risky than vegan respondents did. The difference in risk perception between the two groups was statistically significant (see results).

Since many studies confirm the importance of risk perception for healthy behaviors [39–43], this result has implications for the development of public health interventions. Because perceived risks directly reduce the likelihood of adoption, it seems important to communicate not only the documented health benefits but also possible risks which might apply to a vegan diet in contrast to other diets. From the TPB perspective [26], these differing risk perceptions imply variations in attitudes toward the behavior, which is a central determinant of behavioral intention.

### Vegan diet and vulnerable groups

The scientific literature has demonstrated that a vegan diet can offer numerous health benefits (see introduction). However, the question of associated health risks, particularly for vulnerable groups, remains subject to medical debate.

Kiely [44] suggests that such diets, especially for young children, pose a risk of nutritional deficiencies, thus necessitating substantial planning, expert guidance, and supplementation. Conversely, Jakše, Fras and Fidler Mis [45] maintain that skeptical views frequently stem from reliance on outdated or unrepresentative studies, advocating for a re-evaluation applying identical criteria to both vegan and omnivorous diets. The low certainty of evidence found in a recent systematic review [46] further underscores the critical need for robustly designed future investigations in this domain. This need is exemplified by the methodological issues observed in an umbrella review on the association between plant- vs. meat-predominant diets and depression, which specifically cites a high reliance on observational studies and a lack of measurement for dietary adherence and quality [47].

The present article’s objective is not to analyze the complex medical data regarding the risks of a vegan diet for vulnerable groups, but rather to establish psychological and behavioral starting points for the development of public health interventions. Instead, the requirement for a comprehensive presentation of all known health risks – encompassing both vegan and omnivorous diets – and outlining effective mitigation strategies should be met by these subsequent intervention frameworks.

Regarding organizational guidelines, both the German Nutrition Society (DGE) and the Academy of Nutrition and Dietetics (A.N.D.) concur that a vegan diet must be properly planned to be nutritionally adequate. A difference exists, however, in the recommendations for vulnerable groups: The DGE [21] takes no explicit stance for or against a vegan diet for these populations. In contrast, the A.N.D [22]. deems a vegetarian, including vegan, diet to be appropriate across all stages of the life cycle, provided it is properly planned. It is essential that local authorities developing behavioral interventions address the health risks of both dietary styles in light of current medical literature. The significant difference in risk perception found in our study between vegan and omnivorous participants underlines the importance of this statement.

### Information sources, perceived advantages, and supplementation

These differences in risk perception between vegan and omnivore participants may be linked to differences in their perceived nutritional knowledge and information access. Omnivorous participants reported a higher reliance on the internet and traditional media (newspapers, magazines, and television), whereas vegan participants favored online social networks and books.

Regarding the perceived usefulness of sources, the vegan group rated books as significantly more helpful. Conversely, the omnivorous group rated magazines, friends/acquaintances, family, scientific studies, physicians, and nutritionists as significantly more useful. These findings, though exhibiting small effect sizes, can be useful for public health interventions, as they suggest addressing the two groups through different information channels. Importantly, both groups considered general internet sources (excluding social networks) highly helpful.

The two groups reported distinct advantages and disadvantages associated with their respective diets. Vegan respondents primarily cited health, ethical, and environmental benefits (e.g., animal welfare, climate) and perceived drawbacks mainly related to implementation challenges (e.g., high costs, need for supplementation, social conflict).

Omnivorous respondents also reported health benefits (including eating a balanced diet with all nutrients) but saw disadvantages in negative health impacts (unbalanced diet), and negative impacts on climate, environment, and animals. Both groups reported the enjoyment of eating as a key advantage. This indicates that both groups associate positive health outcomes with their chosen diet while simultaneously being aware of its potential negative health effects. As seen in the risk perception analysis, this awareness highlights the need for public health interventions to adequately address both the risks and benefits of vegan and omnivorous diets.

When questioned about specific health indicators, the vegan group perceived their diet to have a notably greater positive health impact than the omnivorous group. The perceived benefit included reduced risk of diabetes, cardiovascular problems, and cancer, alongside improved vitamin and mineral supply. This statistically significant difference in benefit perception aligns with the actual documented health advantages of a vegan diet but should be viewed in light of recent research emphasizing the importance of diet quality over diet type alone. For example, studies suggest that diet quality, rather than the diet type itself (plant-based or omnivorous), may be an important factor in psychological well-being, correlating significantly with lower depressive symptoms [48]. Specifically, within the plant-based community, a high-quality diet has been found to be protective against depressive symptoms, especially in non-depressed individuals [49].

The finding that the vegan group reported improved vitamin and mineral supply as a notable advantage of their diet (see Table 5) appears to conflict with the fact that more vegan participants (69%) than omnivorous participants (28%) reported taking supplements. A similar pattern was observed in a study by Weikert et al. [50], in which almost all vegan participants and one third of the omnivorous participants reported consuming supplements in the past 4 weeks. However, it is possible that vegan respondents consider supplementation an integral part of their chosen diet, with increased engagement and monitoring leading to a perception of improved overall nutritional management. In contrast, for non-adherents, the perception of a potential requirement for these risk mitigation strategies may be viewed as a significant implementation challenge. Such implementation challenges directly affect PBC – an individual’s assessment of how easy or difficult it is to perform a specific behavior. PBC is a core component of TPB [26], which has a direct influence on actual behavior [27]. Public health interventions could therefore account for these differing supplementation behaviors. Further studies are warranted to explore the exact nature of the perceived intake and actual supplement use.

### Limitations

The present study offers valuable insights into the psychological factors of dietary choice but is subject to several limitations that should be considered for interpretation and future research.

#### Sampling, design, and external validity

A primary limitation lies in the study’s cross-sectional design, which only allows for the examination of associations at a single point in time. This prevents the derivation of any causal inference between the identified psychological factors and actual dietary behavior. Furthermore, the online survey design and its non-representative sample may restrict the generalizability of the findings. This methodology is susceptible to self-selection bias, which may result in an overrepresentation of motivated individuals. This, in turn, could introduce potential confounds, for instance, a possible overestimation of positive attitudes in the vegan group. Additionally, the study’s focus on participants in Germany introduces an element of cultural specificity, meaning the findings may not be directly transferable to other cultures or healthcare systems. The limitation of matching only on age and gender also means relevant sociodemographic differences (e.g., income or education) may remain as unobserved confounding factors.

#### Measurement and self-report bias

The study’s reliance on self-reported data is a constraint. For example, nutritional knowledge was measured only through self-assessed information levels rather than objective assessments. Consequently, we could not verify the accuracy of the vegan participants’ perceived knowledge advantage, making these findings subject to social desirability bias. General limitations of online surveys apply; notably, the risk of inaccurate or inattentive responses from participants in uncontrolled settings remains, even with quality control measures in place.

#### Scope and theoretical asymmetry

The scope of the investigation presents an asymmetry that affects the derivation of comprehensive intervention strategies. While we closely examined motivations for the adoption of a vegan diet and their challenges, we did not explore the psychological factors driving the maintenance of an omnivorous diet. Such insights would beneficial for developing interventions to promote dietary change. Similarly, the risk perception assessment was asymmetrical, focusing predominantly on the perceived risks of a vegan diet. A symmetrical investigation, which also assessed the perceived risks associated with an omnivorous diet (e.g., in relation to circulatory diseases), would have yielded a more comprehensive basis for developing a public health intervention.

#### Data depth on risk mitigation

Our data regarding diet for vulnerable groups (e.g., during pregnancy or childhood) were limited to the reported dietary style. We did not collect information on risk minimization strategies, such as consistent supplementation or routine medical consultations. Since many vegan respondents reported low-risk perceptions for these groups, it remains unclear whether this belief is rooted in confidence in such mitigation strategies or maybe a lack of awareness. Consequently, this gap in data impedes the derivation of optimally tailored educational content in this regard.

## Conclusion

This study systematically analyzed psychological and social factors associated with the adoption and maintenance of a vegan diet. Our results confirm previous findings while offering novel insights that could be beneficial for the development of public health interventions.

The motivation to adopt a vegan diet appears strongly linked to ethical, health, and ecological concerns. Notably, the frequent role of video-based media as a trigger suggests a potential for digital platforms to influence the reflection on dietary change. Regarding the social environment, networks with other vegans seem to be supportive.

A central finding is the statistically significant difference in perceived health risks between the two groups, with omnivorous participants consistently reporting higher risk perception, particularly for vulnerable groups. This difference in perception may act as a barrier to adoption. Furthermore, the divergence in preferred information channels between the groups suggests a multi-modal communication approach may be advantageous.

Finally, the higher rate of supplementation among vegan participants, coupled with the perception of improved nutritional supply, suggest that supplementation might be integrated as a responsible component of dietary management.

These key observations directly align with the core constructs of the Theory of Planned Behavior (TPB [26]),. The differing risk and benefit perceptions between the groups reflect varying attitudes; the social influence patterns relate to subjective norms; and the identified challenges like supplementation point towards factors affecting PBC.

The results suggest several areas where public health strategies could be informed by these associations:

1. Risk and Benefit Communication: Public health interventions may benefit from specifically addressing the differing risk perceptions. This could involve providing scientifically supported information that clarifies the potential health benefits and potentially required risk mitigation strategies (e.g., proper planning and supplementation) relevant to both vegan and omnivorous diets.
2. Potential of Digital Media: Given the frequent association between video content and dietary triggers, social media and digital platforms may be a suitable means for reaching target groups, consistent with the observed role of video content in dietary triggers.
3. Targeted Audience Focus: Intervention planning may benefit from considering the identified social factors and the precise definition of the target group. The finding that children in vegan households are a significant secondary audience supports the rationale for including family-centric nutritional guidance.
4. Channel Specificity: The distinct preferences for information sources (e.g., books vs. television) suggests a potential requirement for interventions to utilize different information channels to maximize reach across different audiences.
5. Addressing Supplementation: The higher rate of supplementation among vegan participants may be a perceived barrier by non-adherents. Interventions could address this by providing scientifically substantiated information about the potential requirement for supplementation when following a vegan diet.

As this investigation relies on a cross-sectional design, the reported findings are associational and do not permit causal inference. Future research would therefore benefit from employing longitudinal or experimental designs to rigorously test the impact of interventions that build upon the psychological determinants identified in this study.

In conclusion, the successful promotion of a well-planned vegan diet may be facilitated by interventions that strategically utilize digital and visual content. These interventions could also employ targeted, evidence-based communication to address existing risk-benefit perceptions. To maximize efficacy, these efforts could strategically target the key psychological determinants identified by the Theory of Planned Behavior – namely Attitudes, Subjective Norms, and Perceived Behavioral Control – and acknowledge the interplay between individual health beliefs and social influence.

## Supplementary Information

Supplementary Material 1

## Data Availability

The datasets used and/or analyzed during the current study are available from the corresponding author on reasonable request.
