# Supplementary material for: Comparing diet-related attitudes, perceptions, and behaviors of vegan and omnivorous adults: results from a cross-sectional survey study in Germany
Source: BMC Public Health. 2025 Dec 23;25:4279. doi: 10.1186/s12889-025-25528-5 (PMC12723827; doi:10.1186/s12889-025-25528-5)
Supplement: Supplementary file 1 — Supplementary Material 1 [file 12889_2025_25528_MOESM1_ESM.docx]

**Questionnaire**

**Project: Vegan diet**

**Project no: 955**

**Method: CAWI (Panel)**

**Target group: Vegans + omnivores**

**Number of cases: 1,550 (750 vegans, 800 omnivores)**

**Overview:**

[Module I - Screening](#_Toc528065946)

[Module A - Motives for vegan](#_Toc528065947) diet

[Module B - Social environment, pregnancy and children](#_Toc528065948)

[Module C - Knowledge](#_Toc528065949)

[Module D -](#_Toc528065950) Dietary habits

[Module E - Health behaviour](#_Toc528065951)

Module S - Sociodemographics

Module I - Screening

**Question Intro**

Thank you for taking part in this survey. Our survey is about lifestyle and health. We also ask questions about your personal behaviour. If you do not wish to answer certain questions, you have the option of clicking on the "don't know/no response" option at any point in the questionnaire. Participation in the survey and answering each individual question is voluntary. The data is collected anonymously and is only used for scientific purposes. It is not possible to draw any conclusions about you as an individual.

**Question S1 - Age**

How old are you? Please enter your age in the field.

1. |__|__|__| years

99 No answer

**Question S1_A - Age**

1. 16-29 years
2. 30-39 years
3. 40-49 years
4. 50-59 years
5. 60-69 years
6. 70-79 years
7. 80-89 years
8. 90-99 years

**Question S2 - Gender**

You are ...

1. male
2. female
3. diverse

99 don't know / no answer

**Question I1a - Diet**

What is your diet? Please tick all that apply to you.

1. vegan, i.e. no animal food products such as dairy produce, eggs, meat and fish
2. vegetarian, i.e. with dairy produce and/or eggs and/or fish but **no** meat products
3. mixed diet, i.e. food of both animal (meat and other products) and plant origin
4. wholefood diet, i.e. lots of fruit, vegetables and grains, little meat and sweets
5. gluten-free, i.e. without food that contains gluten
6. lactose-free, i.e. without food containing lactose
7. calorie-reduced
8. low-carb i.e. with a reduction in carbohydrates
9. Paleo, i.e. orientated towards the Stone Age diet

98 other, namely _______________________

99 don't know / no answer

**Question I1b - Diet**

Which of the following three dietary groups would you most likely categorise yourself as?

1. vegan, i.e. no animal food products such as dairy produce, eggs, meat and fish
2. vegetarian, i.e. with dairy produce and/or eggs and/or fish but **no** meat products
3. mixed diet, i.e. food of animal (meat and other products) and plant origin

Module A - Motives for vegan diet

Filter: Vegans [I1a = 1 OR I1b = 1].

**Question A1 - Diet prior to vegan diet**

How did you eat before you went vegan?

1. vegetarian, no meat and no sausage**, but fish**
2. vegetarian, no meat, no sausage and **no fish**
3. mixed diet, i.e. food of animal (meat and other products) and plant origin

99 don't know / no answer

Filter: Vegans [I1a = 1 OR I1b = 1].

**Question A2 - Duration of veganism**

How long have you been following a vegan diet? If you do not know exactly,
please estimate.

1. less than 1 year
2. 1 to less than 2 years
3. 2 to less than 3 years
4. 3 to under 4 years
5. 4 to under 5 years
6. 5 to under 10 years
7. 10 to 15 years
8. longer than 15 years

99 don't know / no answer

Filter: Vegans [I1a = 1 OR I1b = 1].

**Question A3a - Motives for vegan nutrition**

Which of the following was your **main motive** for deciding to go vegan?

1. ethical reasons, e.g. animal rights, animal husbandry
2. health reasons, e.g. food intolerance, improved well-being
3. ecological reasons, e.g. environmental protection, sustainability
4. religious and / or spiritual reasons
5. social and / or political reasons

98 other, namely: _________________

99 don't know / no answer

Filter: Vegans, if main motive 'health reasons' applies [(I1a = 1 OR I1b = 1) AND A3a = 2].

**Question A3b - Health reasons for vegan diet**

What specific health reasons were crucial for your decision to follow a vegan diet? Please tick all that apply to you.

1. allergy / allergies
2. high blood pressure
3. chronic inflammatory disease (e.g. rheumatoid arthritis, chronic intestinal inflammation,
   chronic skin disease)
4. diabetes
5. cancer
6. food intolerance (e.g. lactose intolerance)
7. obesity

98 other, namely: _____________

99 don't know / no answer

Filter: Vegans [I1a = 1 OR I1b = 1].

**Question A4a - Key experience for switching to vegan diet**

Was there a key experience that led you to switch to a vegan diet?

1. yes
2. no

99 don't know / no answer

Filter: Vegans with key experience [(I1a = 1 OR I1b = 1) AND A4a = 1].

**Question A4b - Key experience for switching to vegan diet**

Which of the following key experiences was crucial for you to switch to a vegan diet? Please tick only the answer that applies to you the most.

1. health problems
2. documentary film about animal rights, animal husbandry
3. documentary film about vegan diet
4. radio documentary about animal rights, animal husbandry
5. radio documentary about vegan diet
6. documentary literature on animal rights, animal husbandry, e.g. book, brochure, online article, etc.
7. documentary literature on vegan diet, e.g. book, brochure, online article, etc.
8. pregnancy / breastfeeding

98 other, namely: ____________

99 don't know / no answer

Filter: Vegans [I1a = 1 OR I1b = 1].

**Question A5a - People who influenced your decision to adopt a vegan diet**

Were there one or more people who influenced your decision to follow a vegan diet?

1. yes
2. no

99 don't know / no answer

Filter: Vegans, with influence from another person [(I1a = 1 OR I1b = 1) AND A5a= 1].

**Question A5b - People who influenced the decision to adopt a vegan diet**

How much influence did the following people have on your decision to follow a vegan diet?

1. parents or siblings
2. partner
3. other relatives or children
4. friends or acquaintances
5. flatmates in a shared flat
6. colleagues
7. famous person(s) /celebrity
8. no influence at all
9. partial influence
10. crucial influence

99 don't know / no answer

Filter: Vegans, famous person(s) / celebrity had crucial or partial influence [(I1a = 1 OR I1b = 1) AND A5b (7) = 2-3].

**Question A5c – Famous person(s) / celebrity who exert influence**

Which famous person(s) or celebrity had an influence on your decision to follow a vegan diet?

*Note: Please write your answer in the text box.*

_____________________________

99 No answer

Module B - Social environment, pregnancy and children

Filter: Vegans [I1a = 1 OR I1b = 1].

**Question B1 - Vegans in the social environment**

Now we are interested in the diet of the people in your environment. Are there people in your environment who follow a vegan diet? Please tick everything that applies to you.

1. yes, in my household
2. yes, family members who do not live in my household
3. yes, friends, acquaintances who do not live in my household
4. yes, namely _____________________
5. no

99 don't know / no answer

**Question B2 - Children under the age of 18 in the household**

Do children under the age of 18 live in your household?

1. yes
2. no

99 don't know / no answer

Filter: Children under the age of 18 in the household [B2 = 1].

**Question B3 - Children in the household**

How many children of the following ages live in your household? Please enter the number of children. Please enter 0 if no child of the corresponding age group lives in your household.

1. I__I child(ren) aged up to 12 months: |__|
2. I__I child(ren) aged 1-2 years: |__|
3. I__I child(ren) aged 3-6 years: |__|
4. I__I child(ren) aged 7-10 years: |__|
5. I__I child(ren) aged 11-13 years: |__|
6. I__I child(ren) aged 14-17 years: |__|

99 don't know / no answer

Filter: Vegans with children up to 12 months in the household [B3(1)> 0].

**Question B4a - Diet of children, up to 12 months**

How do you feed your child or children up to the age of 12 months at home?

1. vegan
2. vegetarian, no meat and no sausage, **but fish**
3. vegetarian, no meat, no sausage and **no fish**
4. mixed diet, i.e. food of animal (meat and other products) and plant origin

99 don't know / no answer

Filter: Vegans with children aged 1-2 in the household [B3(2)> 0].

**Question B4b - Diet of children, 1-2 years old**

How do you feed your child or children aged 1-2 years at home?

1. vegan
2. vegetarian, no meat and no sausage, **but fish**
3. vegetarian, no meat, no sausage and **no fish**
4. mixed diet, i.e. food of animal (meat and other products) and plant origin

99 don't know / no answer

Filter: Vegans with children aged 3-6 in the household [B3(3)> 0].

**Question B4c - Diet of children, 3-6 years old**

How do you feed your child or children aged 3-6 at home?

1. vegan
2. vegetarian, no meat and no sausage**, but fish**
3. vegetarian, no meat, no sausage and **no fish**
4. mixed diet, i.e. food of animal (meat and other products) and plant origin

99 don't know / no answer

Filter: Vegans with children aged 7-10 in the household [B3(4)> 0].

**Question B4d - Diet of children, 7-10 years old**

How do you feed your child or children aged 7-10 at home?

1. vegan
2. vegetarian, no meat and no sausage, **but fish**
3. vegetarian, no meat, no sausage and **no fish**
4. mixed diet, i.e. food of animal (meat and other products) and plant origin

99 don't know / no answer

Filter: Vegans with children aged 11-13 in the household [B3(5)> 0].

**Question B4e - Diet of children, 11-13 years old**

How do you feed your child or children aged 11-13 at home?

1. vegan
2. vegetarian, no meat and no sausage, **but fish**
3. vegetarian, no meat, no sausage and **no fish**
4. mixed diet, i.e. food of animal (meat and other products) and plant origin

99 don't know / no answer

Filter: Vegans with children in the household [B3(6)> 0].

**Question B4f - Diet of children, 14-17 years old**

How do you feed your child or children aged 14-17 at home?

1. vegan
2. vegetarian, no meat and no sausage**, but fish**
3. vegetarian, no meat, no sausage and **no fish**
4. mixed diet, i.e. food of animal (meat and other products) and plant origin

99 don't know / no answer

Filter: Vegan and female [(I1a = 1 OR I1b = 1) AND S2 = 2].

**Question B5a - Pregnancy in the last 10 years**

Have you been pregnant in the last 10 years?

1. yes
2. no

99 don't know / no answer

Filter: Vegan and pregnant in the last 10 years [(I1a = 1 OR I1b = 1) AND B5a = 1].

**Question B5b - Diet during pregnancy**

What was your diet during pregnancy?

1. vegan
2. vegetarian, no meat and no sausage, **but fish**
3. vegetarian, no meat, no sausage and **no fish**
4. mixed diet, i.e. food of animal (meat and other products) and plant origin

99 don't know / no answer

Module C - Knowledge

**Question C1a - Sources of information**

Now we are interested in your knowledge about nutrition.

Do you actively inform yourself about nutrition?

1. yes
2. no

99 don't know / no answer

Filter: If you actively inform yourself about nutrition [C1a = 1].

**Question C1b - Sources of information (open)**

Which sources do you use to actively inform yourself about nutrition?

Note: Please write your answer in the text field.

________________________

99 No answer

Filter: If you actively inform yourself about nutrition [C1a = 1].

**Question C1c - Sources of information (closed-ended)**

To what extent do you agree with the statement that the following sources can provide you with helpful information about nutrition?

1. Internet forums
2. social networks on the Internet
3. other sources on the Internet
4. magazines
5. books
6. friends or acquaintances
7. family
8. scientific studies, e.g. from the German Nutrition Society
9. physicians
10. nutritionists
11. strongly disagree
12. tend to disagree
13. partly / partly
14. tend to agree
15. strongly agree

99 don't know / no answer

**Question C2 - Subjective assessment of knowledge about the advantages and disadvantages of vegan diet**

Regardless of your current diet, how well informed do you feel about the advantages and disadvantages of a **vegan diet**?

1. very well
2. well
3. partly / partly
4. less well
5. not well at all

99 don't know / no answer

**Question C4 - Subjective assessment of knowledge about the advantages and disadvantages of a mixed diet**

Regardless of your current diet: How well informed do you feel about the advantages and disadvantages of a **mixed diet**?

1. very well
2. well
3. partly / partly
4. less well
5. not well at all

99 don't know / no answer

**Question C6a - Health benefits of your own diet**

Do you see advantages to your current diet?

1. yes

2 no

99 don't know / no answer

Filter: Respondents who see benefits in their diet [C6a = 1].

**Question C6b - Health benefits of your own diet (open)**

What advantages do you see?

Note: Please write your answer in the text field.

_________________________________

99 No answer

**Question C6c - Health disadvantages of your current diet**

Do you see any disadvantages to your current diet?

1. yes

2 no

99 don't know / no answer

Filter: Respondents who see disadvantages in their diet [C6c = 1].

**Question C6d - Health disadvantages of your own diet (open)**

What disadvantages do you see?

Note: Please write your answer in the text field.

_________________________________

99 No answer

**Question C7a - Health effects of your own diet (closed-ended)**

To what extent do you agree or disagree with the following statements about the impact of your current diet on your health?

1. The probability of diabetes increases.
2. The probability of cardiovascular disease increases.
3. Metabolism deteriorates.
4. Oxygen transport deteriorates.
5. Cholesterol levels fall.
6. The probability of cancer decreases.
7. The supply of vitamins and minerals improves.
8. It becomes easier to achieve or maintain a healthy body weight.
9. strongly disagree
10. tend to disagree
11. partly / partly
12. tend to agree
13. strongly agree

99 don't know / no answer

Filter: Vegans [I1a = 1 OR I1b = 1].

**Question C7b - Subjective assessment of health status after switching to vegan diet**

Has switching to a vegan diet had an impact on your health or well-being?

1 yes

2 no

99 don't know / no answer

Filter: Vegan, with influence on health [(I1a = 1 OR I1b = 1) AND C7b = 1].

**Question C8a - Specific improvement of health status**

What impact has the change in diet had on your health or well-being?

Note: Please write your answer in the text field.

__________________________________

1. No answer

**Question C8b - Risk of vitamin/mineral deficiency**

In your opinion, is there a risk of vitamin or mineral deficiency in your current diet without taking regular food supplements?

1 yes

2 no

99 don't know / no answer

Filter: If risk of vitamin / mineral deficiency is perceived [C8b = 1].

**Question C9a - Vitamin or mineral deficiency**

For which vitamin or mineral could there be a deficiency due to your current diet? Please tick all the nutrients for which you think there may be a deficiency.

1. calcium
2. iron
3. iodine
4. magnesium
5. phosphorus
6. vitamin A
7. vitamin B1
8. vitamin B12 (cobalamin)
9. vitamin B2
10. vitamin B6
11. vitamin B9 (folic acid)
12. vitamin C
13. vitamin D
14. vitamin E
15. vitamin H (biotin)
16. vitamin K
17. zinc

98 other, namely: ______________

96 none of the above

99 don't know / no answer

**Question C9b - Groups of people for whom a vegan diet is risky**

In your opinion, are there groups of people for whom a vegan diet is associated with health risks?

1 yes

2 no

99 don't know / no answer

Filter: vegan diet not suitable for everyone according to previous question [C9b = 1].

**Question C9c - Groups of people for whom a vegan diet is risky**

In your opinion, for which groups of people is a vegan diet associated with health risks?

*Note: Please write your answers in the text field.*

__________________________________

99 No answer

**Question C9d - Groups of people for whom a vegan diet is risky**

In the following, we will name groups of people that you may not have mentioned. To what extent do you agree with the statement that a vegan diet is associated with health risks for the following groups of people? Please tick all that apply in your opinion.

1. infants (up to 12 months)
2. toddlers (1-3 years)
3. children (4-12 years)
4. adolescents (13-18 years)
5. pregnant women
6. breastfeeding women
7. elderly people aged 65 and over
8. chronically ill people
9. people between the ages of 19 and 64
10. strongly disagree
11. tend to disagree
12. partly / partly
13. tend to agree
14. strongly agree

99 don't know / no answer

Filter: Vegans and omnivores who fully or partially agree with the statements [C9d (1) = 3-5].

**Question C9e - Risk mitigation for vulnerable groups (up to 12 months)**

What measures can be taken to mitigate the health risks of a vegan diet in **infants (up to 12 months)**? Please tick all that apply in your opinion.

1. through regular medical check-ups / counselling
2. by taking food supplements
3. by eating enriched food
4. by attending nutritional counselling

98 other, namely ________________________

96 none of the above

99 don't know / no answer

Filter: Vegans and mixed dieters who fully or partially agree with the statements [C9d (2) = 3-5].

**Question C9f - Risk mitigation for vulnerable groups (1-3 years)**

What measures can be taken to mitigate the health risks of a vegan diet for
**toddlers (1-3 years)**? Please tick all that apply in your opinion.

1. through regular medical check-ups / counselling
2. by taking food supplements
3. by eating enriched food
4. by attending nutritional counselling

98 other, namely ________________________

96 none of the above

99 don't know / no answer

Filter: Vegans and mixed dieters who fully or partially agree with the statements [C9d (3) = 3-5].

**Question C9g - Risk mitigation for vulnerable groups (4-12 years)**

What measures can be taken to mitigate the health risks of a vegan diet in **children**
**(4-12 years)**? Please tick all that apply in your opinion.

1. through regular medical check-ups / counselling
2. by taking food supplements
3. by eating enriched food
4. by attending nutritional counselling

98 other, namely ________________________

96 none of the above

99 don't know / no answer

Filter: Vegans and mixed dieters who fully or partially agree with the statements [C9d (4) = 3-5].

**Question C9h - Risk mitigation for vulnerable groups (13-18 years)**

What measures can be taken to mitigate the health risks of a vegan diet in
**adolescents (13-18 years)**? Please tick all that apply in your opinion.

1. through regular medical check-ups / counselling
2. by taking food supplements
3. by eating enriched food
4. by attending nutritional counselling

98 other, namely ________________________

96 none of the above

99 don't know / no answer

Filter: Vegans and omnivores who fully or partially agree with the statements [C9d (5) = 3-5].

**Question C9i - Risk mitigation for vulnerable groups (Pregnant women)**

What measures can be taken to mitigate the health risks of a
vegan diet in **pregnant women**? Please tick all that apply in your opinion.

1. through regular medical check-ups / counselling
2. by taking food supplements
3. by eating enriched food
4. by attending nutritional counselling

98 other, namely ________________________

96 none of the above

99 don't know / no answer

Filter: Vegans and omnivores who fully or partially agree with the statements [C9d (6) = 3-5].

**Question C9j - Risk mitigation for vulnerable groups (Breastfeeding women)**

What measures can be taken to mitigate the health risks of a vegan diet for **breastfeeding women**? Please tick all that apply in your opinion.

1. through regular medical check-ups / counselling
2. by taking food supplements
3. by eating enriched food
4. by attending nutritional counselling

98 other, namely ________________________

96 none of the above

99 don't know / no answer

Filter: Vegans and mixed dieters who fully or partially agree with the statements [C9d (7) = 3-5].

**Question C9k - Risk mitigation for vulnerable groups (people aged 65 and over)**

What measures can be taken to mitigate the health risks of a vegan diet for **people aged 65 and over**? Please tick all that apply in your opinion.

1. through regular medical check-ups / counselling
2. by taking food supplements
3. by eating enriched food
4. by attending nutritional counselling

98 other, namely ________________________

96 none of the above

99 don't know / no answer

Filter: Vegans and mixed dieters who fully or partially agree with the statements [C9d (8) = 3-5].

**Question C9l - Risk mitigation for vulnerable groups (chronically ill people)**

What measures can be taken to mitigate the health risks of a vegan diet for
**chronically ill people**? Please tick all that apply in your opinion.

1. through regular medical check-ups / counselling
2. by taking food supplements
3. by eating enriched food
4. by attending nutritional counselling

98 other, namely ________________________

96 none of the above

99 don't know / no answer

Filter: Vegans and mixed food eaters who fully or partially agree with the statements [C9d (9) = 3-5].

**Question C9m - Risk mitigation for vulnerable groups (People between 19 and 64 years of age)**

What measures can be taken to mitigate the health risks of a vegan diet for **people aged between 19 and 64**? Please tick all that apply in your opinion.

1. through regular medical check-ups / counselling
2. by taking food supplements
3. by eating enriched food
4. by attending nutritional counselling

98 other, namely ________________________

96 none of the above

99 don't know / no answer

Module D - Dietary habits

**Question D1a - Self-assessment of frequency of consumption**

We are now interested in other aspects of your diet. How often do you eat the following foods?

(1) Ready meals

(2) processed food

(3) home-prepared food

1. never
2. less than once a month
3. about 1-3 times a month
4. about once a week
5. several times a week
6. daily

99 don't know / no answer

**Question D1b - Self-assessment of frequency of fruit and vegetable consumption**

How often do you eat vegetables, salad and / or fruit? One portion corresponds to about a handful of fruit,
vegetables or salad.

1. never
2. less than 1 portion per week
3. less than 1 portion per day
4. 1-2 portions per day
5. 3-4 portions per day
6. 5 and more portions per day

99 don't know / no answer

Filter: Omnivore [I1 = 3].

**Question D2 - Meat consumption of omnivores**

What do you estimate: How much meat and/or sausage products do you eat in a typical week?

For a better estimate: an average portion of meat weighs about **150 g.** A slice of
sausage weighs about **20 g**. Seven slices of sausage correspond to one portion of meat.

1. less than 1 portion of meat per week (150 g)
2. 1 to less than 2 portions of meat per week (from 150 to less than 300 g)
3. 2 to less than 4 portions of meat per week (from 300 to less than 600 g)
4. 4 to less than 6 portions of meat per week (from 600 to less than 900 g)
5. at least 6 portions of meat per week (from 900 g)

99 don't know / no answer

**Question D3a - Food supplements**

Do you take food supplements? Explanation: Food supplements are products
intended to supplement general nutrition. They contain concentrated
nutrients such as vitamins, minerals and trace elements, amino acids, but also dietary fibres, plants or herbal extracts. They are available in dosed form, e.g. as tablets, capsules, coated tablets
but also as powders and liquids.

1. yes
2. no

99 don't know / no answer

Filter: If food supplements are taken [D3a = 1].

**Question D3b - Food supplements**

Which food supplements do you take? Please tick all the nutrients that you take
in dosed form (e.g. as tablets, capsules, coated tablets, powder or liquids).

1. calcium
2. iron
3. iodine
4. magnesium
5. phosphorus
6. vitamin A
7. vitamin B1
8. vitamin B12 (cobalamin)
9. vitamin B2
10. vitamin B6
11. vitamin B9 (folic acid)
12. vitamin C
13. vitamin D
14. vitamin E
15. vitamin H (biotin)
16. vitamin K
17. zinc

98 other, namely: ______________

99 don't know / no answer

Filter: If certain food supplement is taken [D3b = 1].

**Question D3ba - Food supplement frequency**

How often do you take **calcium**?

1. less than once a month
2. about 1-3 times a month
3. about once a week
4. several times a week
5. daily

99 don't know / no answer

Filter: If certain food supplement is taken [D3b = 2].

**Question D3bb - Food supplement frequency**

How often do you take **iron**?

1. less than once a month
2. about 1-3 times a month
3. about once a week
4. several times a week
5. daily

99 don't know / no answer

Filter: If certain food supplement is taken [D3b = 3].

**Question D3bc - Food supplement frequency**

How often do you take **iodine**?

1. less than once a month
2. about 1-3 times a month
3. about once a week
4. several times a week
5. daily

99 don't know / no answer

Filter: If certain food supplement is taken [D3b = 4].

**Question D3bd - Food supplement frequency**

How often do you take **magnesium**?

1. less than once a month
2. about 1-3 times a month
3. about once a week
4. several times a week
5. daily

99 don't know / no answer

Filter: If certain food supplement is taken [D3b = 5].

**Question D3be - Food supplements Frequency**

How often do you take **phosphorus**?

1. less than once a month
2. about 1-3 times a month
3. about once a week
4. several times a week
5. daily

99 don't know / no answer

Filter: If certain food supplement is taken [D3b = 6].

**Question D3bf - Food supplements Frequency**

How often do you take **vitamin A**?

1. less than once a month
2. about 1-3 times a month
3. about once a week
4. several times a week
5. daily

99 don't know / no answer

Filter: If certain food supplement is taken [D3b = 7].

**Question D3bg - Food supplement frequency**

How often do you take **vitamin B1**?

1. less than once a month
2. about 1-3 times a month
3. about once a week
4. several times a week
5. daily

99 don't know / no answer

Filter: If certain food supplement is taken [D3b = 8].

**Question D3bh - Food supplement frequency**

How often do you take **vitamin B12 (cobalamin)**?

1. less than once a month
2. about 1-3 times a month
3. about once a week
4. several times a week
5. daily

99 don't know / no answer

Filter: If certain food supplement is taken [D3b = 9].

**Question D3bi - food supplement frequency**

How often do you take **vitamin B2**?

1. less than once a month
2. about 1-3 times a month
3. about once a week
4. several times a week
5. daily

99 don't know / no answer

Filter: If certain food supplement is taken [D3b = 10].

**Question D3bj - Food supplement frequency**

How often do you take **vitamin B6**?

1. less than once a month
2. about 1-3 times a month
3. about once a week
4. several times a week
5. daily

99 don't know / no answer

Filter: If certain food supplement is taken [D3b = 11].

**Question D3bk - Food supplements Frequency**

How often do you take **folic acid (vitamin B9)**?

1. less than once a month
2. about 1-3 times a month
3. about once a week
4. several times a week
5. daily

99 don't know / no answer

Filter: If certain food supplement is taken [D3b = 12].

**Question D3bl - Food supplements Frequency**

How often do you take **vitamin C**?

1. less than once a month
2. about 1-3 times a month
3. about once a week
4. several times a week
5. daily

99 don't know / no answer

Filter: If certain food supplement is taken [D3b = 13].

**Question D3bm - Food supplement frequency**

How often do you take **vitamin D**?

1. less than once a month
2. about 1-3 times a month
3. about once a week
4. several times a week
5. daily

99 don't know / no answer

Filter: If certain food supplement is taken [D3b = 14].

**Question D3bn - Food supplement frequency**

How often do you take **vitamin E**?

1. less than once a month
2. about 1-3 times a month
3. about once a week
4. several times a week
5. daily

99 don't know / no answer

Filter: If certain food supplement is taken [D3b = 15].

**Question D3bo - food supplement frequency**

How often do you take **vitamin H (biotin)**?

1. less than once a month
2. about 1-3 times a month
3. about once a week
4. several times a week
5. daily

99 don't know / no answer

Filter: If certain food supplement is taken [D3b = 16].

**Question D3bp - Food supplement frequency**

How often do you take **vitamin K**?

1. less than once a month
2. about 1-3 times a month
3. about once a week
4. several times a week
5. daily

99 don't know / no answer

Filter: If certain food supplement is taken [D3b = 17].

**Question D3bq - Food supplement frequency**

How often do you take **zinc**?

1. less than once a month
2. about 1-3 times a month
3. about once a week
4. several times a week
5. daily

99 don't know / no answer

Filter: If food supplements are taken [D3a = 1].

**Question D3br - Food supplements due to chronic illness**

Do you take one or more food supplements due to a chronic illness
(e.g. rheumatoid arthritis, chronic inflammatory bowel disease, chronic skin disease)?

1. yes
2. no

99 don't know / no answer

Module E - Health behaviour

**Question E1 - Subjective assessment of health status**

Now we are interested in your health status. How would you describe your current health status?

1. very poor
2. rather poor
3. partly / partly
4. rather good
5. very good

99 don't know / no answer

Question E3a - Alcohol consumption yes / no

How often do you drink alcohol?

1. never
2. about 1 time per month
3. 2-4 times a month
4. 2-3 times a week
5. 4 times or more per week

99 don't know / no answer

Filter: If consumption of alcoholic beverages [E3a = 2-5].

Question E3b - Number of alcoholic drinks

If you drink alcohol in a day, how many alcoholic drinks do you typically drink?

For a better estimate: One glass of alcohol corresponds to about

0.33 litres of beer

0.25 litres of wine or sparkling wine

0.02 litres of spirits.

1. 1 or 2 litres
2. 3 or 4
3. 5 or 6 litres
4. 7 or 8 litres
5. 9 or more

99 don't know / no answer

Filter: If consumption of alcoholic beverages [E3a = 2-5].

Question E3c - Number of glasses drunk

How often have you drunk 6 or more alcoholic drinks in one day in the last year?

For a better estimate: One glass of alcohol corresponds to approx.

0.33 litres of beer

0.25 litres of wine or sparkling wine

0.02 litres of spirits.

1. never
2. less than 1 time per month
3. 1 time per month
4. 1 time per week
5. daily or almost daily

99 don't know / no answer

Question E4a - Survey of current smoking status

Now we are interested in your smoking behaviour. Do you currently smoke cigarettes - even if only occasionally?

For a better assessment: By regular we mean

At least 1 cigarette per day, *or*

At least 5 cigarettes per week, *or*

*At least 1 packet of cigarettes per month for at least 6 months*

1. yes
2. no, I used to smoke regularly, but no longer do so
3. no, I have **never** smoked regularly

99 don't know / no answer

Filter: Smoker [E4a= 1].

Question E4b - Cigarette smoker regularly Number / day

How much do you usually smoke at the moment? Please enter the number of cigarettes in the field.

1 number of cigarettes per day |__|__|__|

99 don't know / no answer

Module S – Sociodemographics

**Question S3a – Height**

Finally, a few questions about you. How tall are you?

1. Your height in cm: |__|__|__|

99 don't know / no answer

**Question S3b – Body weight**

What is your current weight? If you don’t know, please estimate.

1. Your current body weight (in kilograms): |__|__|__|

99 don't know / no answer

**Question S4 – Highest general education school leaving certificate**

What is your highest level of general education qualification? Please select one.

1. I am still a student
2. Did not obtain a school leaving certificate
3. Hauptschulabschluss (Lower secondary school leaving certificate)
4. Realschulabschluss (Intermediate school leaving certificate)
5. Polytechnic Secondary School (DDR/East Germany) with completion of 8^th^ or 9^th^ grade
6. Polytechnic Secondary School (DDR/East Germany) with completion of 10^th^ grade
7. Fachhochschulreife (Higher technical college entrance qualification / Diploma allowing access to Universities of Applied Sciences)
8. General or subject-specific higher education entrance qualification / Abitur (General or vocational upper secondary school, including the former EOS, also with vocational training)
9. Abitur obtained through the second education route (Second-Chance Education)
10. Another school leaving certificate, namely: _________________________
11. don't know / no answer

**Question S5 – Highest vocational or higher education qualification**

Which vocational qualifications do you possess? Please select all that apply.

1. Currently in vocational training (Vocational preparation year, apprentice, trainee/intern, student)
2. Student and currently attending a vocational secondary school, technical college, or similar institution
3. No vocational qualification and not currently in vocational training
4. Completed vocational apprenticeship (Lehre)
5. Completed vocational school-based training (e.g., Vocational school, Commercial school, Preparatory sevice for the intermediate civil service in public administration)
6. Completed training at a Fachschule (Technical/Specialist School) of the DDR (East Germany)
7. Completed training at a Specialized, Master, Technical School, or Vocational/Professional Academy
8. Completed a Bachelor’s degree at a University (of Applied Sciences)
9. University of Applied Sciences Degree (e.g., Diplom, Master)
10. University Degree (e.g., Diplom, Magister, State Examination, Master)
11. Doctorate
12. Other professional qualification, namely: ____________________

99 don't know / no answer

**Question S6 – Persons in household**

How many people permanently live in your household (including yourself)? Please enter the number.

I__I__I Person(s)

99 don't know / no answer

**Question S8 – Income**

**What was your net income last month, i.e., your wages or salary after the deduction of taxes and social security contributions?**

1. Less than 500 Euros
2. 500 Euros up to less than 1,000 Euros
3. 1,000 Euros up to less than 1,500 Euros
4. 1,500 Euros up to less than 2,000 Euros
5. 2,000 Euros up to less than 3,000 Euros
6. 3,000 Euros up to less than 4,000 Euros
7. 4,000 Euros and more

99 don't know / no answer

**Question S9 – Federal State**

Which Federal State do you live in?

1. Baden-Württemberg
2. Bavaria
3. Berlin
4. Brandenburg
5. Bremen
6. Hamburg
7. Hessen
8. Mecklenburg-Vorpommern
9. Lower Saxony
10. North Rhine-Westphalia
11. Rhineland-Palatinate
12. Saarland
13. Saxony
14. Saxony-Anhalt
15. Schleswig-Holstein
16. Thuringia

99 don't know / no answer

Question S10 – Size of locality

What is the population size of the town/community you live in?

1 Under 5,000 inhabitants

2 5,000 up to less than 20,000 inhabitants

3 20,000 up to less than 100,000 inhabitants

4 100,000 up to less than 500,000 inhabitants

5 Over 500,000 inhabitants

99 don't know / no answer

Question S11 – Citizenship

What is/are your citizenship(s)?

1. German citizenship
2. Other citizenship, namely: ________________

99 don't know / no answer

This concludes the questionnaire. Thank you for your participation.
